# Supplementary material for: TMEM147 aggravates the progression of HCC by modulating cholesterol homeostasis, suppressing ferroptosis, and promoting the M2 polarization of tumor-associated macrophages
Source: J Exp Clin Cancer Res. 2023 Oct 28;42:286. doi: 10.1186/s13046-023-02865-0 (PMC10612308; doi:10.1186/s13046-023-02865-0)
Supplement: Supplementary file 1 — Supplementary Material 1 [file 13046_2023_2865_MOESM1_ESM.pdf]

Certificate of Elsevier Language Editing Services

The following article was edited by Elsevier Language Editing Services:

TMEM147 aggravates the progression of HCC by modulating cholesterol homeostasis, suppressing ferroptosis and promoting M2 polarization of TAMs

Ordered by:

Jingjing Huang

Estimated Delivery date:

2023-09-28

Order reference:

ASLEPLUS0457927
