# Supplementary material for: TMEM147 aggravates the progression of HCC by modulating cholesterol homeostasis, suppressing ferroptosis, and promoting the M2 polarization of tumor-associated macrophages
Source: J Exp Clin Cancer Res. 2023 Oct 28;42:286. doi: 10.1186/s13046-023-02865-0 (PMC10612308; doi:10.1186/s13046-023-02865-0)
Supplement: Supplementary file 2 — Supplementary Material 2 [file 13046_2023_2865_MOESM2_ESM.docx]

**Supplementary Table S1. Detailed clinicopathologic characteristics of the HCC patients.**

| Features | TMEM147 staining | | *P* value |
| --- | --- | --- | --- |
|  | Positive(n=68)  n(%) | Low/negative(n=37)  n(%) |  |
| Age(years) |  |  | 0.549(*P* >0.05) |
| ≥60 | 38(55.88%) | 21(56.76%) |  |
| <60 | 30(44.12%) | 16(43.24%) |  |
| Gender |  |  | 0.287(*P* >0.05) |
| Male | 42(61.76%) | 20(54.05%) |  |
| Female | 26(38.23%) | 17(45.95%) |  |
| Histological differentiation | |  |  |
| Well | 20(29.41%) | 24(64.86%) | **0.000458(*P <0.05*)** |
| Poor | 48(70.59%) | 13(35.14%) |  |
| TNM stage |  |  |  |
| I~II | 22(32.35%) | 24(64.86%) | **0.001312*(P <0.05)*** |
| Ⅲ~Ⅳ | 46(67.65%) | 13(35.14%) |  |
| AFP (μg /L) |  |  |  |
| ≤20 | 44(64.71%) | 21(56.76%) | 0.276(*P* >0.05） |
| >20 | 24(35.29%) | 16(43.24%) |  |

**Supplementary Table S2. List of primers of qRT-PCR used in this study.**

| **gene** | **Forward Primer** | **Reverse Primer** |
| --- | --- | --- |
| TMEM147 | CTGCTCAGGTCTGGATGATAAC | GTGGACGAAGGTCTCCATAAC |
| DHCR7 | GCTGCAAAATCGCAACCCAA | GCTCGCCAGTGAAAACCAGT |
| IDI2 | GACTGGGTTGACAGGCGTC | GTCGGCACCAATAACCTTATCAT |
| MSMO1 | TGCTTTGGTTGTGCAGTCATT | GGATGTGCATATTCAGCTTCCA |
| EBP | CTCAGCACCTAAGACTGGACA | ACGACTAAGACCCCTGTGACA |
| FDFT1 | CCACCCCGAAGAGTTCTACAA | TGCGACTGGTCTGATTGAGATA |
| HMGCS1 | GATGTGGGAATTGTTGCCCTT | ATTGTCTCTGTTCCAACTTCCAG |
| SQLE | GGCATTGCCACTTTCACCTAT | GGCCTGAGAGAATATCCGAGAAG |
| CYP27A1 | CGGCAACGGAGCTTAGAGG | GGCATAGCCTTGAACGAACAG |
| STAT2 | CCAGCTTTACTCGCACAGC | AGCCTTGGAATCATCACTCCC |
| GPX4 | GAGGCAAGACCGAAGTAAACTAC | CCGAACTGGTTACACGGGAA |
| HMOX1 | AAGACTGCGTTCCTGCTCAAC | AAAGCCCTACAGCAACTGTCG |
| SLC7A11 | TCTCCAAAGGAGGTTACCTGC | AGACTCCCCTCAGTAAAGTGAC |
| NCOA4 | GAGGTGTAGTGATGCACGGAG | GACGGCTTATGCAACTGTGAA |
| NRF2 | TCAGCGACGGAAAGAGTATGA | CCACTGGTTTCTGACTGGATGT |
| CP | GGGCCATCTACCCTGATAACA | TTAAAGGTCCGATGAGTCCTGA |
| CD206 | TCCGGGTGCTGTTCTCCTA | CCAGTCTGTTTTTGATGGCACT |
| ARG1 | GTGGAAACTTGCATGGACAAC | AATCCTGGCACATCGGGAATC |
| CPT1A | TCCAGTTGGCTTATCGTGGTG | TCCAGAGTCCGATTGATTTTTGC |
| PPARγ | GGGATCAGCTCCGTGGATCT | TGCACTTTGGTACTCTTGAAGTT |

**Supplementary Table S3. List of primers of qRT-PCR used in this study.**

| **Antibody** | **Company** |
| --- | --- |
| TMEM147 | Abcam |
| DHCR7 | Abcam |
| IDI2 | Abcam |
| MSMO1 | Abcam |
| EBP | Abcam |
| FDFT1 | Abcam |
| HMGCS1 | Abcam |
| SQLE | Abcam |
| CYP27A1 | Abcam |
| STAT2 | Abcam |
| GPX4 | Abcam |
| HMOX1 | Abcam |
| SLC7A11 | Abcam |
| NCOA4 | Abcam |
| NRF2 | Abcam |
| CP | Abcam |
| CD206 | Abcam |
| ARG1 | Abcam |
| CPT1A | Abcam |
| PPARγ | Abcam |
| β-actin | Cell Signaling Technology |
| β-tublin | Cell Signaling Technology |
| CD163 | BD Biosciences |
| HLA-DRα | BD Biosciences |
