# Supplementary figures and images for: TMEM147 aggravates the progression of HCC by modulating cholesterol homeostasis, suppressing ferroptosis, and promoting the M2 polarization of tumor-associated macrophages
Source: J Exp Clin Cancer Res. 2023 Oct 28;42:286. doi: 10.1186/s13046-023-02865-0 (PMC10612308; doi:10.1186/s13046-023-02865-0)

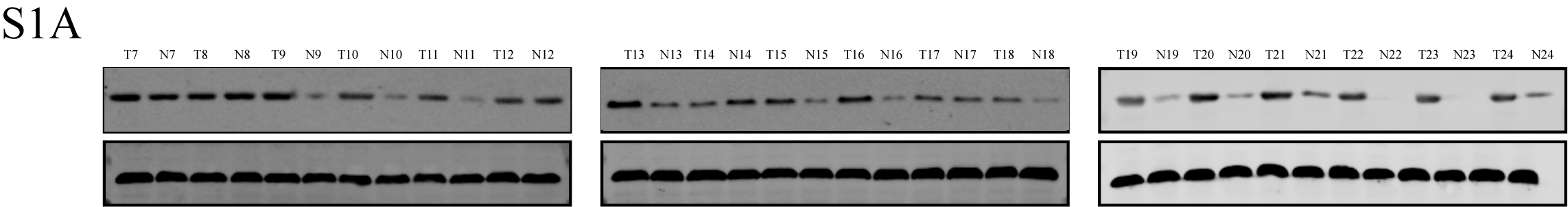

Supplement: Supplementary file 3 — Supplementary Material 3 [file 13046_2023_2865_MOESM3_ESM.tif]

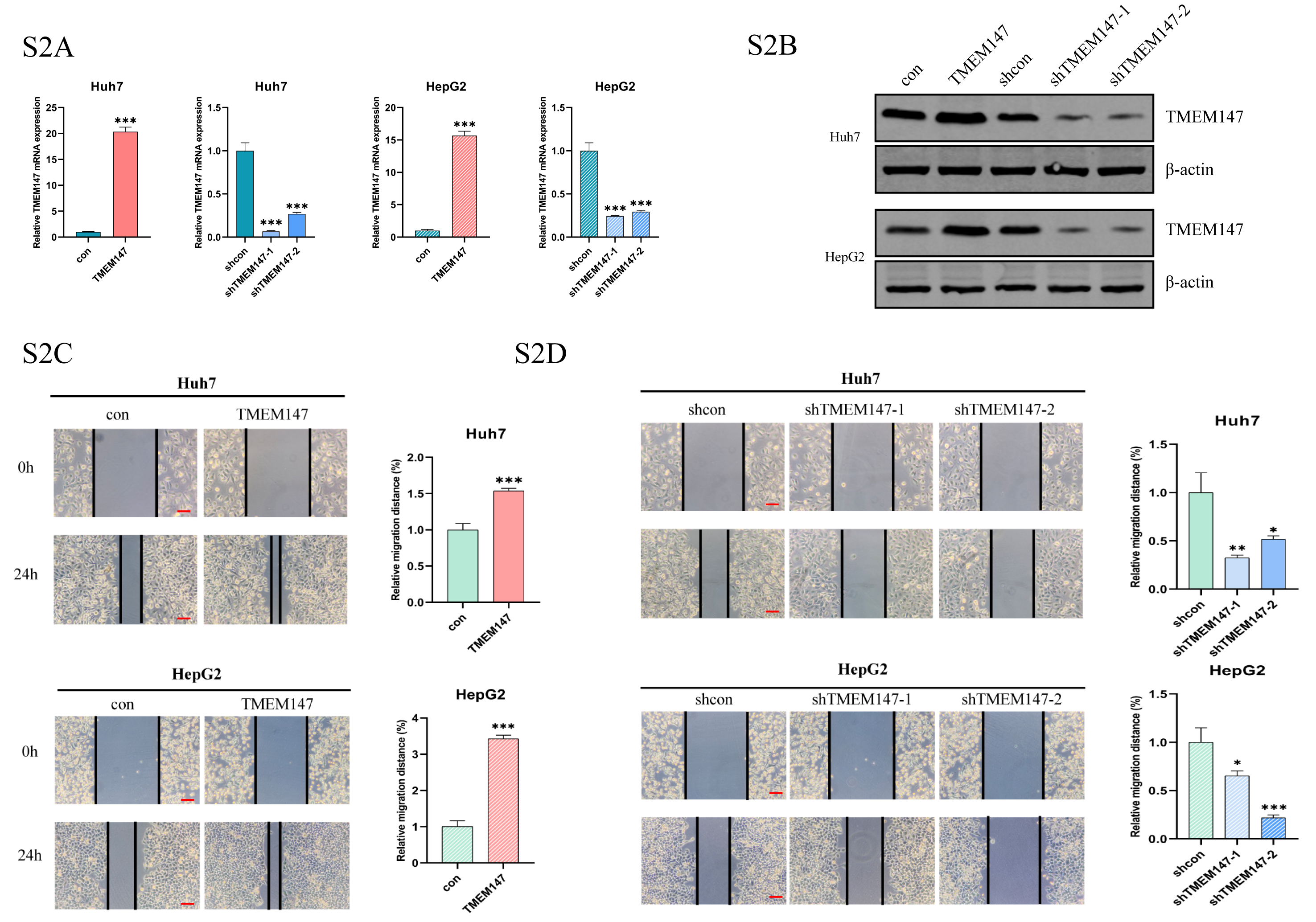

Supplement: Supplementary file 4 — Supplementary Material 4 [file 13046_2023_2865_MOESM4_ESM.tif]

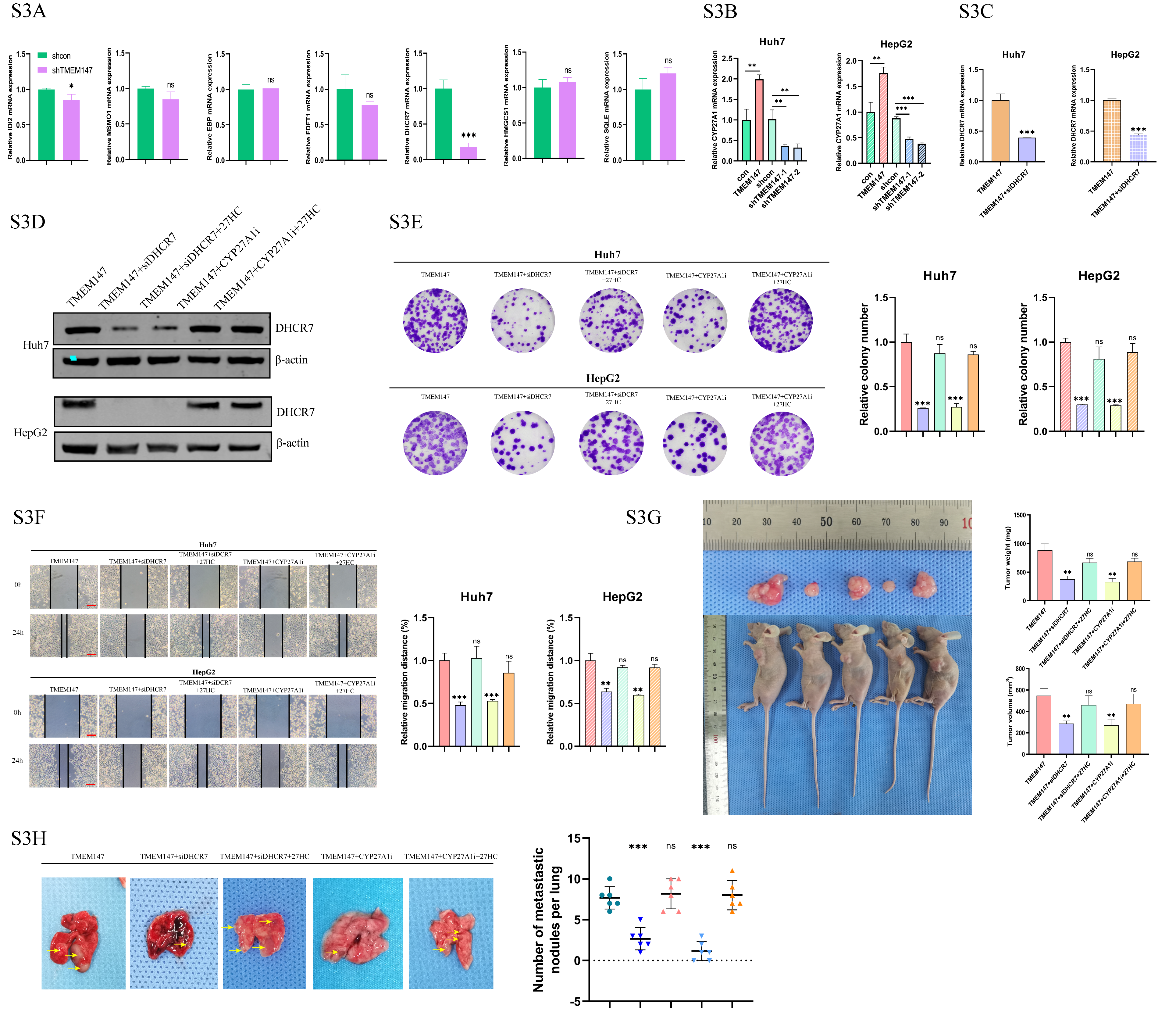

Supplement: Supplementary file 5 — Supplementary Material 5 [file 13046_2023_2865_MOESM5_ESM.tif]

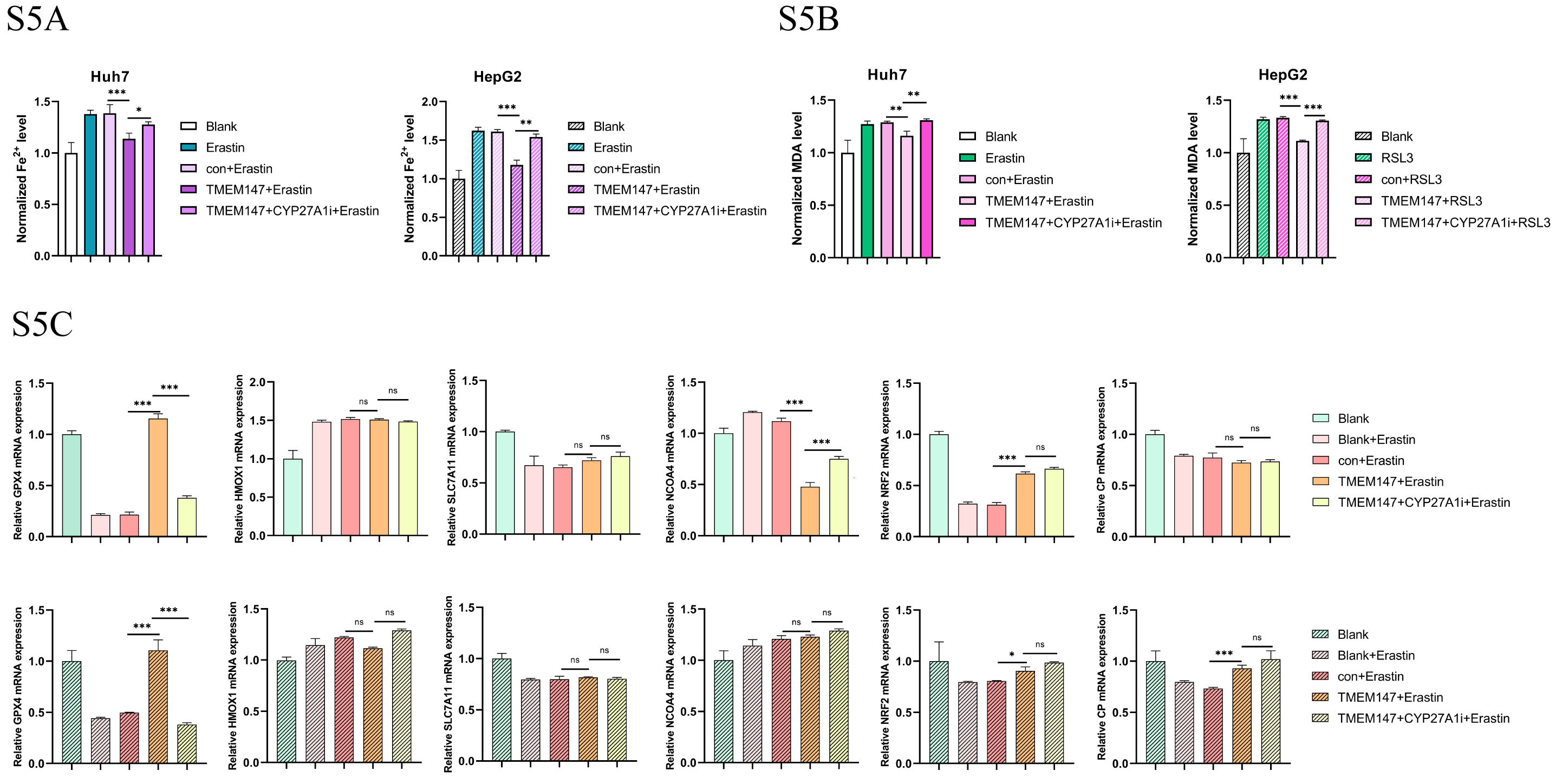

Supplement: Supplementary file 6 — Supplementary Material 6 [file 13046_2023_2865_MOESM6_ESM.tif]

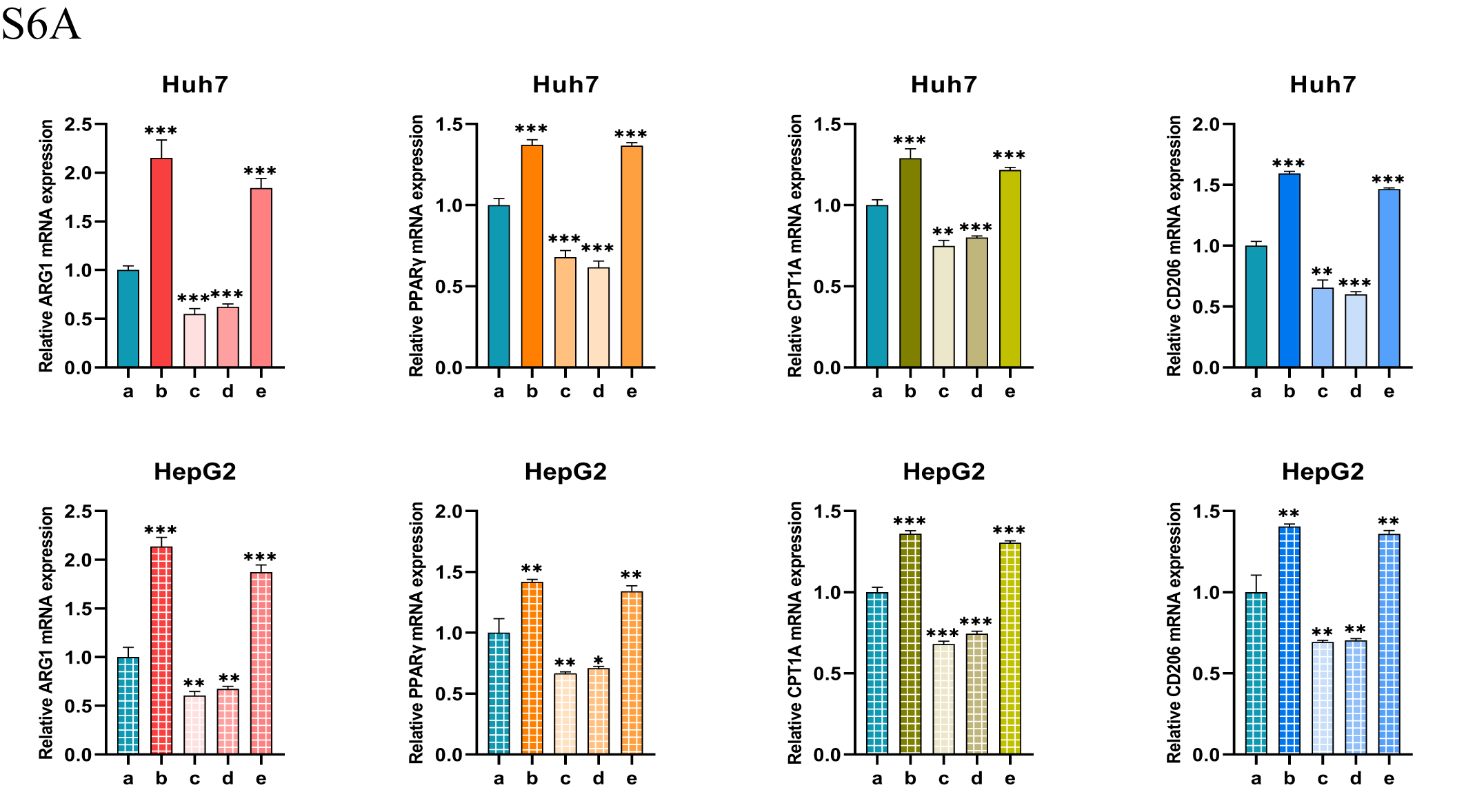

Supplement: Supplementary file 7 — Supplementary Material 7 [file 13046_2023_2865_MOESM7_ESM.tif]

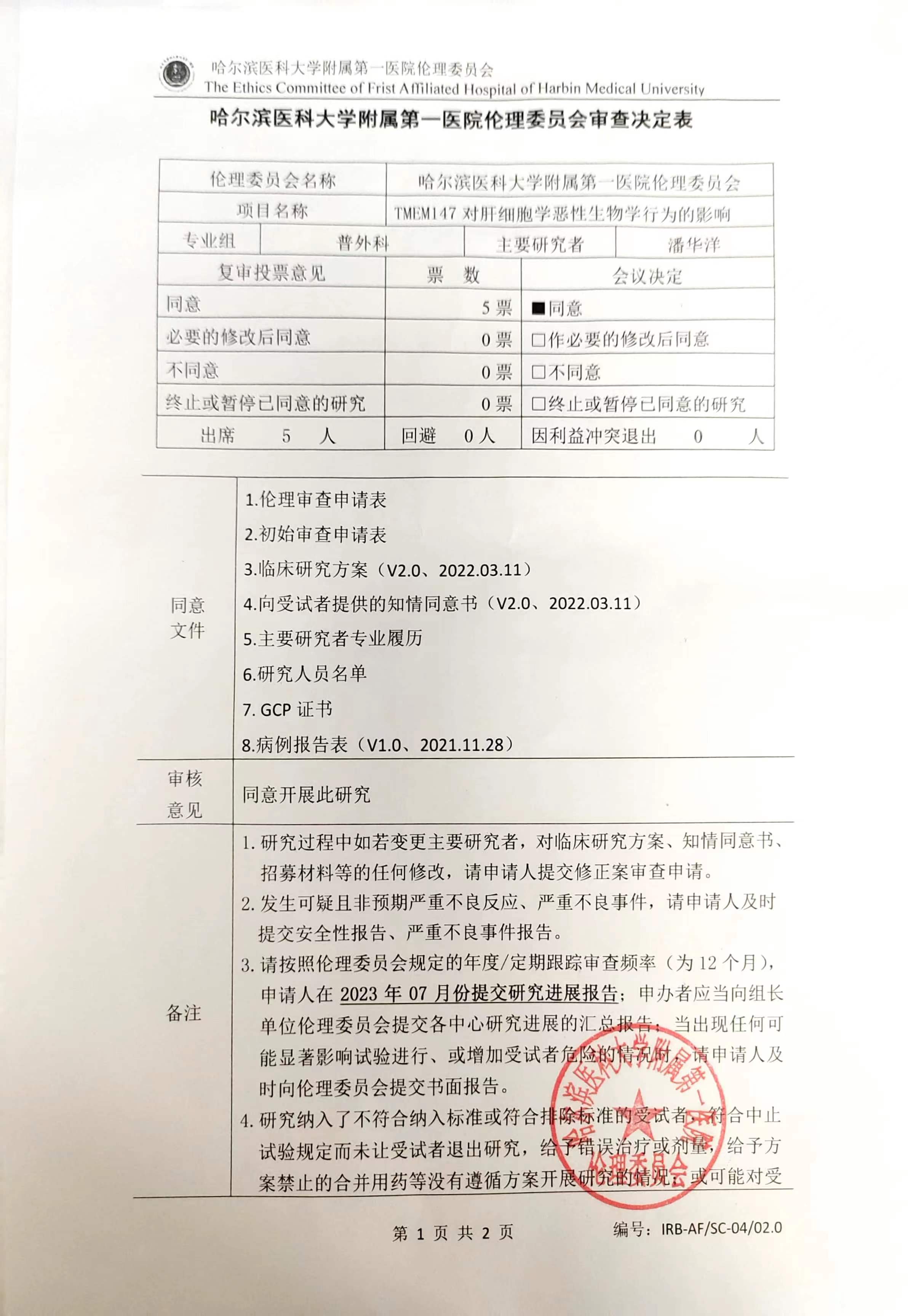

Supplement: Supplementary file 9 — Supplementary Material 9 [file 13046_2023_2865_MOESM9_ESM.jpg]

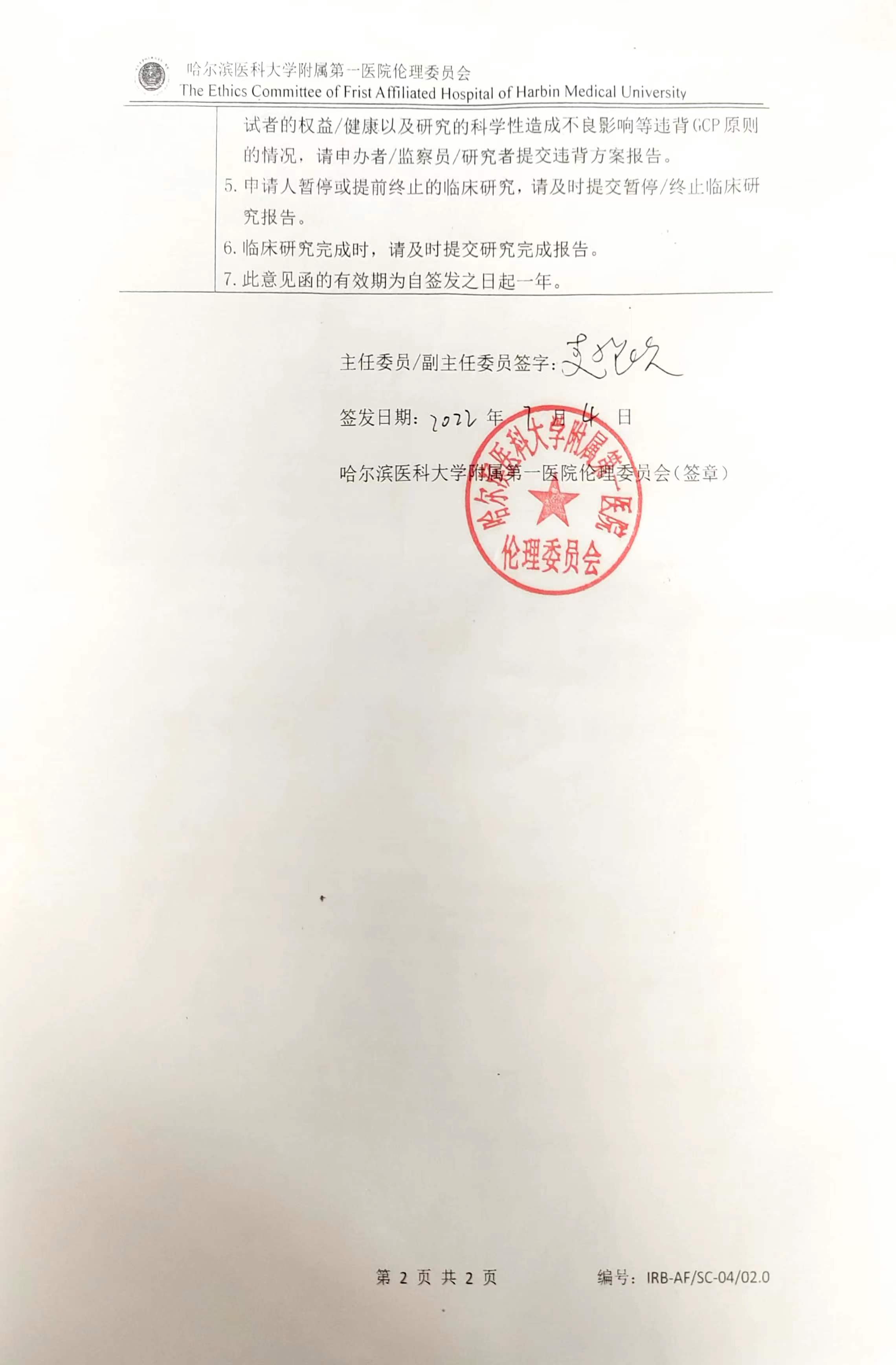

Supplement: Supplementary file 10 — Supplementary Material 10 [file 13046_2023_2865_MOESM10_ESM.jpg]

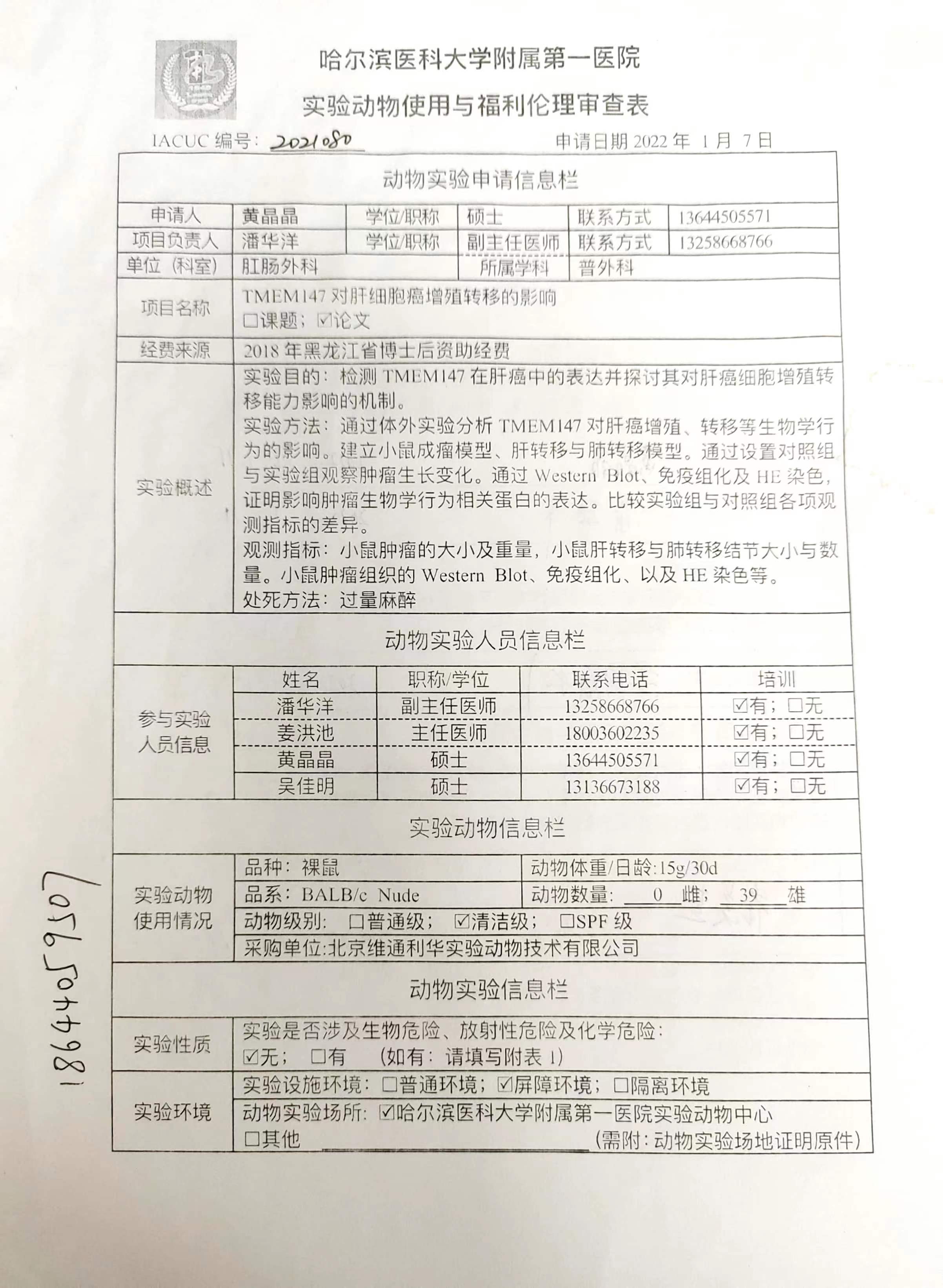

Supplement: Supplementary file 11 — Supplementary Material 11 [file 13046_2023_2865_MOESM11_ESM.jpg]

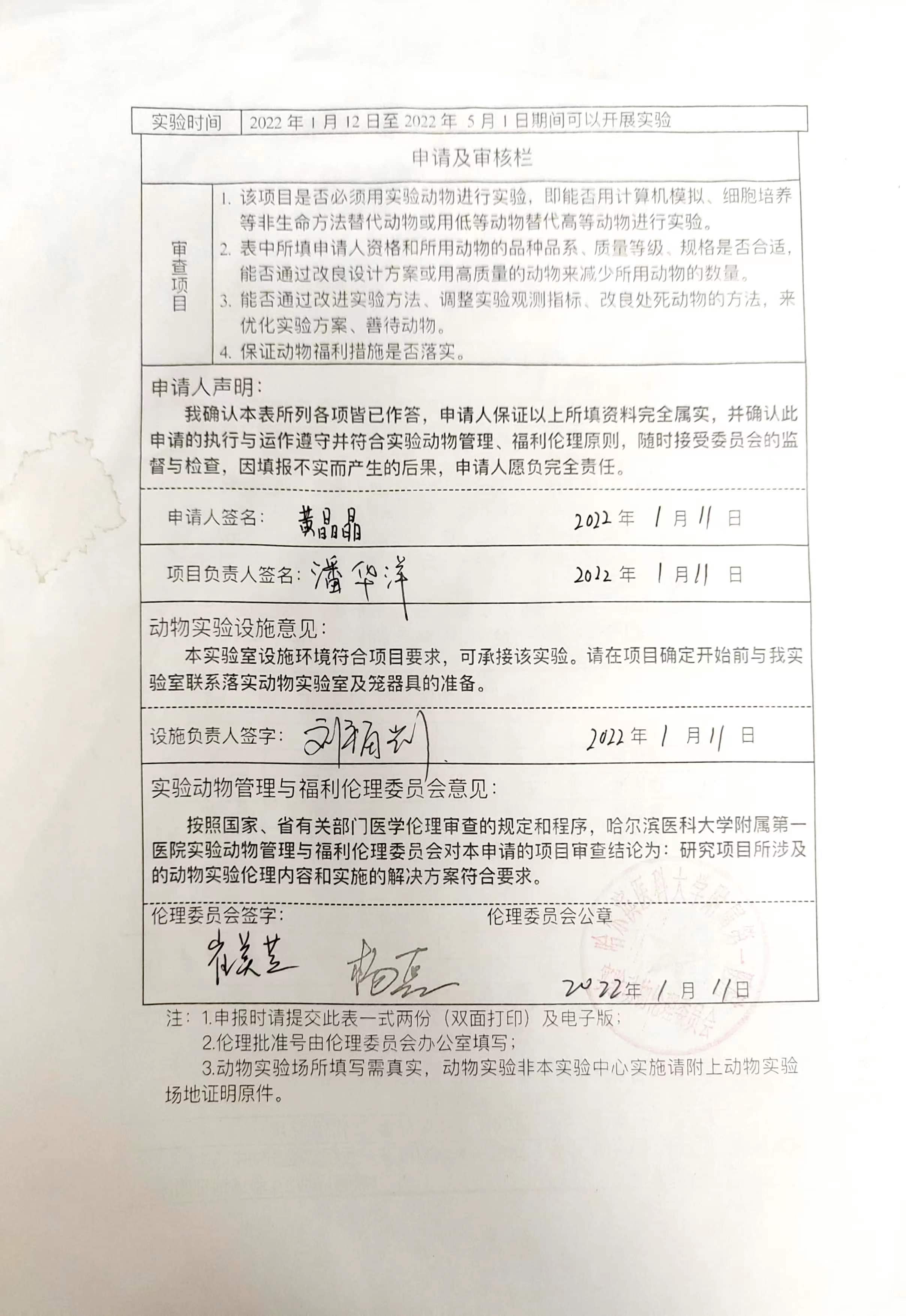

Supplement: Supplementary file 12 — Supplementary Material 12 [file 13046_2023_2865_MOESM12_ESM.jpg]
