# Supplementary material for: TMEM147 aggravates the progression of HCC by modulating cholesterol homeostasis, suppressing ferroptosis, and promoting the M2 polarization of tumor-associated macrophages
Source: J Exp Clin Cancer Res. 2023 Oct 28;42:286. doi: 10.1186/s13046-023-02865-0 (PMC10612308; doi:10.1186/s13046-023-02865-0)
Supplement: Supplementary file 13 — Supplementary Material 13 [file 13046_2023_2865_MOESM13_ESM.docx]

**Title page:**

**TMEM147 aggravates the progression of HCC by modulating cholesterol homeostasis, suppressing ferroptosis, and** **promoting the M2 polarization of tumor-associated macrophages**

Jingjing Huang^1,2†^, Huayang Pan^1,2†*^, Jing Sun^1,2†^, Jiaming Wu^1,2†^, Qiyue Xuan^1,2^, Jinge Wang^5^, Shanjia Ke^1,2^, Shounan Lu^1,2^, Zihao Li^1,2^, Zhigang Feng^1,2,3^, Yongliang Hua^1,4^, Qingan Yu^1,2^，Bing Yin^1,2^, Baolin Qian^1,2^, Menghua Zhou^1,2^, Yanan Xu^1,2^, Miaoyu Bai^1,2^, Yingmei Zhang^1,2^, Yaohua Wu^1,2^, Yong Ma^1,2^, Hongchi Jiang^1,2^ ^*^, Wenjie Dai^1,2^ ^*^

^1^Key Laboratory of Hepatosplenic Surgery, Ministry of Education, the First Affiliated Hospital of Harbin Medical University, Harbin, China.

^2^Department of General Surgery, the First Affiliated Hospital of Harbin Medical University, Harbin, China.

^3^The First Department of General Surgery, Affiliated Hospital of Inner Mongolia Minzu University, Tongliao, China.

^4^Department of Pediatric Surgery, the First Affiliated Hospital of Harbin Medical University, Harbin, China.

^5^School of Public Health, Harbin Medical University, Harbin, China

^†^These authors contributed equally: Jingjing Huang, Huayang Pan, Jing Sun, Jiaming Wu. ✉email: panhuayang66@hrbmu.edu.cn; [jianghongchi@hotmail.com](mailto:jianghongchi@hotmail.com); daiwenjiehmu@163.com.

**Abstract**

**Background:** The endoplasmic reticulum (ER) regulates critical processes, including lipid synthesis, which are affected by transmembrane proteins localized in the ER membrane. One such protein, transmembrane protein 147 (TMEM147), has recently been implicated for its role in hepatocellular carcinoma (HCC) tumorigenesis; however, the mechanisms remain unclear. We investigated the role of TMEM147 in HCC and the underlying mechanisms.

**Methods:** TMEM147 expression was examined in human HCC cells and adjacent non-tumorous tissues using quantitative reverse transcription-polymerase chain reaction, western blotting, and immunohistochemistry. *In vitro* and *in vivo* studies were conducted to investigate the impact of TMEM147 on the progression of HCC. Proteins interacting with TMEM147 were identified via RNA-seq, immunoprecipitation, and mass spectrometry analyses. Lipidomic analysis and enzyme-linked immunosorbent assay (ELISA) were employed to determine and analyze cholesterol and 27-hydroxycholesterol (27HC) contents. Extensive experimental techniques were used to study ferroptosis in HCC cells. The fatty acid content of macrophages affected by TMEM147 was quantified using ELISA. Macrophage phenotypes were determined using immunofluorescence assay and flow cytometric analysis.

**Results:** TMEM147 mRNA and protein levels were increased in HCC cells, and the increased TMEM147 expression was associated with a poor survival. TMEM147 promoted tumor cell proliferation and metastases *in vitro* and *in vivo*. The protein was found to interact with the key enzyme 7-dehydrocholesterol reductase (DHCR7), which affected cellular cholesterol homeostasis and increased the extracellular levels of 27HC in HCC cells. TMEM147 also promoted the expression of DHCR7 by enhancing the activity of signal transducer and activator of transcription 2. 27HC expression upregulated glutathione peroxidase 4 in HCC, leading to ferroptosis resistance and promotion of HCC proliferation. HCC cell-derived 27HC expression increased the lipid metabolism in macrophages and activated peroxisome proliferator-activated receptor-γ signaling, thereby activating M2 macrophage polarization and promoting HCC cell invasion and migration.

**Conclusions:** Our results indicate that TMEM147 confers ferroptosis resistance and M2 macrophage polarization, which are primarily dependent on the upregulation of cellular cholesterol homeostasis and 27HC secretion, leading to cancer growth and metastasis. These findings suggest that the TMEM147/STAT2/DHCR7/27HC axis in the tumor microenvironment may serve as a promising therapeutic target for HCC.

**Keywords:** Hepatocellular carcinoma, TMEM147, Cholesterol metabolites, Ferroptosis, Tumor-associated macrophages

**Background**

Hepatocellular carcinoma (HCC), the primary malignancy of hepatocytes, is the most common liver malignancy and a leading cause of cancer-related deaths worldwide. Although multiple treatment modalities exist, surgical resection has been found to be most effective for early-stage HCC. ^[1]^ However, the prognosis of patients with HCC remains poor because of its propensity for metastatic progression and poor response to systemic therapeutic treatments. Therefore, new diagnostic and therapeutic targets must be identified in order to improve the prognosis of patients with HCC.

Transmembrane Protein 147 (TMEM147) is a small, recently identified, 25 kDa protein that localizes to both the nuclear envelope (NE) and the endoplasmic reticulum (ER) membrane. The ER membrane hosts critical cellular processes, including lipid synthesis.^[2, 3]^ TMEM147 has seven transmembrane domains, each of which fully spans the ER membrane, with the N-terminus facing the ER lumen and the C-terminus facing the cytosolic side of the membrane.^[4]^ The expression of this protein in many tissues suggests that it has broad and diverse physiological roles.^[5]^ The role of TMEM147 has been studied in various human diseases, including prostate cancer,^[6]^ rheumatoid arthritis,^[7]^ and colon cancer^[8]^. Consistent with our findings, TMEM147 levels in colon cancer were considerably higher than those in controls, which suggested that it could serve as a biomarker for the disease. TMEM147-AS1 (lncRNA TMEM147 antisense RNA 1) promotes tumorigenesis in prostate cancer by controlling prostatic carcinoma cell invasion and proliferation. However, its role in HCC has not been explored. Our findings show that TMEM147 plays a crucial role in the progression and prognosis of HCC. Further mechanistic exploration revealed that the downstream target is the gene encoding 7-dehydrocholesterol reductase (DHCR7). DHCR7 catalyzes the conversion of 7-dehydrocholesterol to cholesterol, which is the final step of cholesterol synthesis in the Kandutsch–Russell pathway.^[9]^ Previous research has indicated that DHCR7 plays important roles in several kinds of malignant tumors. For example, elevated DHCR7 expression indicates a poor prognosis for cervical cancer^[10]^; DHCR7 promotes tumorigenesis by activating the PI3K/AKT/mTOR signaling pathway in bladder cancer^[11]^; mutations in DHCR7 are significantly associated with the circulating 25-hydroxyvitamin D concentration and the risk of ovarian cancer development in Europeans^[12]^; proliferation, migration, and invasion of GC cells are reduced by DHCR7 knockdown but increased by DHCR7 overexpression both *in vivo* and *in vitro* ^[13]^. Additionally, researchers have found that DHCR7 is significantly related to colorectal cancer risk.^[14]^ However, the role of DHCR7 in HCC is yet unclear and calls for further research. The present study revealed that DHCR7 promotes the production of cholesterol esters and the downstream metabolite 27-hydroxycholesterol (27HC), which plays an essential part in the role of TMEM147 in progression of HCC. It has been reported that DHCR7 can be regulated by many transcription factors, such as sterol-regulatory element binding protein 1/2 (SREBP),^[15, 16]^ osteogenic transcription factor (Runx2),^[17]^ and E2F transcription factor 1 (E2F1). ^[18]^ We found that the signal transducer and activator of transcription factor 2 (STAT2) was activated by TMEM147, thereby promoting DHCR7 transcription.

Ferroptosis is an intracellular novel form of programmed cell death that involves accumulation of iron-dependent lipid peroxides and is distinct from apoptosis, necrosis, and autophagy. Ferroptosis plays a vital role in the progression of HCC.^[19]^ Multiple signaling pathways dictate the susceptibility of cells to ferroptosis. For instance, the glutathione (GSH)-glutathione peroxidase 4 (GPX4) axis, the main ferroptosis-regulating pathway in mammals, together with the E-cadherin-NF2-Hippo-YAP pathway, which dictates ferroptosis sensitivity, have important implications. Studies have shown that the chronic exposure of breast cancer cells to 27HC increases their resistance to ferroptosis. ^[20]^ However, whether TMEM147, an upstream regulator of 27HC, affects ferroptosis in HCC remains unclear.

Tumor-associated macrophages (TAMs) are the most abundant immune cells in the tumor microenvironment (TME) and play a key role in tumor-associated inflammation. TAMs undergo two forms of activation in response to TME signaling. M1 macrophages are antitumorigenic, whereas M2 macrophages are pro-tumorigenic.^[21]^ Various cancer-derived metabolites, such as phosphatidylserine,^[22]^ succinate,^[23]^ lactate,^[24]^ exosomal,^[25, 26]^ and tumor-secreted protein S (Pros1), can regulate the M2 polarization of TAMs.^[27]^ Recent studies have revealed that 27HC affects cellular functions in the TME, such as those of T cells,^[28]^ osteoclast,^[29]^, myeloid cells^[30]^; however, it is unclear whether 27HC regulates the polarization of TAMs.

We demonstrate for the first time that TMEM147 increases the phosphorylation of the transcription factor STAT2 and promotes the transcription of the downstream gene *DHCR7*, thus contributing to HCC proliferation and metastases. *DHCR7* alters cholesterol homeostasis in HCC by increasing the levels of the downstream metabolite 27HC, which protects HCC cells from ferroptosis via the GPX4 pathway. It can also be discharged into the TME, activating fatty acid oxidation (FAO) metabolic reprogramming in TAM, thereby promoting M2 polarization. M2 polarized TAM can promote HCC invasion and metastasis in a positive feedback manner. Our study demonstrates that TMEM147 can serve as a diagnostic biomarker and a crucial therapeutic target for HCC, and targeting its downstream pathways and metabolites may aid in developing targeted therapeutic strategies for HCC therapy.

**Methods**

**HCC specimens**

Only patients with pathological diagnosis of HCC were included in this study. We collected paired HCC and adjacent non-cancerous liver tissue samples from patients who underwent liver resection at the First Affiliated Hospital of Harbin Medical University between January 2012 and August 2017. Senior pathologists were invited to perform pathological diagnoses on the paraffin sections.

The study was conducted in accordance with the principles of the Declaration of Helsinki and approved by the Research Ethics Committee of the First Affiliated Hospital of Harbin Medical University. All patients provided written informed consent.

**Cell lines and cell culture**

Human HCC cell lines SK-Hep-1, Huh7, HepG2, HCCLM3, and BEL-7402 were obtained from the Chinese Academy of Science (Shanghai, China). The normal liver cell line WRL-68 was obtained from AcceGen (Fairfield, USA). The cell lines were cultured in Dulbecco’s Modified Eagle’s Medium (Gibco, USA) supplemented with 10% fetal bovine serum (FBS, Gibco), 100 U/mL penicillin, and 100 μg/mL streptomycin. Human monocytic leukemia cells, THP-1, were purchased from the American Type Culture Collection (ATCC) and cultured in RPMI 1640 medium containing 10% fetal bovine serum and 100 U/mL penicillin, and 100 μg/mL streptomycin. THP-1 cells were treated with 100 nM phorbol myristate acetate (PMA) (Sigma-Aldrich, USA) for 48 h to induce differentiation into macrophages. All cells were incubated at 37 °C in a humidified atmosphere (5% CO_2_).

**Lentivirus infection**

Lentiviral vectors for human *TMEM147* overexpression (lenti-TMEM147) and downregulation (lenti-TMEM147) and *DHCR7* and *GPX4* downregulation (lenti-sh DHCR7 and lenti-sh GPX4, respectively) were obtained from Hanbio Biotechnology Co. Ltd. (Shanghai). Corresponding empty vectors (lenti-Con and lenti-shcon) were used as negative controls. (Hanbio, Shanghai, China).

The transfection was performed with a multiplicity of infection (MOI) of 10–30 in the presence of polybrene (5 μg/mL). Following lentiviral infection, single-cell clones were selected with 3.5 μg/mL puromycin (Sigma-Aldrich) after 2 weeks of incubation. Stably transfected clones were isolated and used for *in vitro* and *in vivo* experiments.

**Immunohistochemical staining**

The tissue sections were deparaffinized, rehydrated, blocked with 10% normal goat serum, and incubated with the anti-TMEM147 (Abcam, ab97624; 1:200 dilution), anti-4HNE (Abcam, ab46544; 1:250 dilution), and anti-ARG1 (Abcam, abab96183; 1:250 dilution) primary antibodies overnight at 4 °C. The slides were then incubated sequentially, first with a secondary antibody (Vector lab, Burlingame, CA, USA) for 1 h and then with Vectastain Elite ABC reagent (Vector Lab) for 30 min. The tissue sections were stained with diaminobenzidine (DAB kit; Vector Laboratories) and counterstained with hematoxylin (Sigma-Aldrich). The percentage score was defined as follows: 0: 0–5%; 1: 5–25%; 2:, 26–50%; 3: 51–75%; 4: 76–100%; and the staining intensity was defined as 1: weak; 2: moderate; and 3: strong and provided a Multiple index (MI) score (MI = intensity × percentage) as follows: MI = 0, scored as 1; MI = 1–4, scored as 2; MI = 5–8, scored as 3; MI = 9 or 12, scored as 4. Samples with scores ≥ 3 were considered to exhibit high expression, whereas those with scores ≤ 2 were classified as exhibiting low expression.

**Western blotting**

Protein samples extracted from tissues or cells were separated by gel electrophoresis, and transferred onto nitrocellulose membranes (Invitrogen, Carlsbad, USA). Subsequently, the membrane was blocked with 5% skim milk and incubated with primary antibodies overnight at 4 ℃. Finally, the membrane was incubated with IRDye 800CW secondary antibody (LI-COR, USA) (1:10,000 dilution) at room temperature for 1 h, and the Odyssey® Imaging System (LI-COR, USA) was used to visualize and analyze the proteins. Details of the primary antibodies against the target proteins are listed in Additional file 1: Table S3.

**Quantitative reverse transcription-polymerase chain reaction (qRT-PCR)**

Total RNA was isolated from freshly frozen tissue and logarithmically growing cells using the Total RNA Miniprep Kit (Axygen Scientific, Inc., USA) according to the manufacturer’s instructions and reverse-transcribed into cDNA using a qPCR RT Kit (TOYOBO, Shanghai, China) after RNA quantification. qRT-PCR was performed using the THUNDERBIRD SYBR qPCR Mix (TOYOBO, Shanghai, China) on an ABI PRISM 7500HT instrument (Applied Biosystems). The expression level of the target mRNA was normalized to the glyceraldehyde-3-phosphate dehydrogenase (*GAPDH*) expression level and was determined according to the 2^-ΔΔ^*^CT^* method. The primers used are listed in Additional file 1: Table S2.

**Cell counting Kit-8 (CCK-8) and colony formation assays**

1000–1500 stably transfected cells were seeded onto a 96-well plate and cultured for 4 h for attachment. Subsequently, the culture solution was replaced with a solution containing CCK-8 reagent and the optical density (OD) value was determined. Colony formation was assayed by plating 1000 cells in petri dishes with 6 cm diameter after 14 days of culture. The medium was then discarded, and the colonies were fixed with 4% paraformaldehyde (PFA) and stained with 0.5% crystal violet.

**Wound-healing assay**

Stably transfected cells were seeded in a 6-well plate at 30 × 10^4^ cells/well and cultured until cell fusion occurred. A 10-μL pipette tip was used to scratch a straight cut at the bottom of the plate. The floating cells were washed away with phosphate buffered saline (PBS) and cultured in serum-free medium, and wound closure was photographed at 0 and 24 h.

**Transwell migration and invasion assay**

Matrigel-coated (BD Biosciences, Franklin Lakes, NJ, USA) or non-Matrigel-coated Transwell plates were used to examine the invasion and migration abilities of the cells. Cells were inoculated into the upper chamber of the transwell and serum-free medium was added. Normal media was injected into the plate wells. After a 48 h incubation period, cells from the upper layer filter were discarded, and the cells in the bottom layer were fixed, stained, and counted.

**Immunofluorescence (IF) assay**

Cells were seeded on polylysine-coated coverslips, cultured for 24 h, and fixed with 4% paraformaldehyde, followed by 0.1% Triton-X-100 permeabilization. Next, cells were incubated with primary antibodies, secondary antibodies (Invitrogen) and DAPI (Vector Laboratories) in sequence. The images were captured with a camera attached to a microscope.

**Co-immunoprecipitation (IP) assay**

Cells were harvested and then lysed in 500 μL co-IP buffer containing a protease inhibitor cocktail (Sigma-Aldrich). After centrifugation, cell lysates were collected and pre-cleared by incubating with 20 μL immobilized protein A/G beads for 1 h at 4 °C. The beads were then discarded using a magnetic frame, and the lysates incubated with primary antibody or control immunoglobulin (Ig)G on a rotator at 4 °C overnight. On the following day, 20 μL of immobilized protein A/G beads were added to precipitate the protein complex at 4 °C for 4 h. Subsequently, samples were washed five times, the beads boiled in loading buffer, and the proteins were prepared for Western blot as described above.

**Mass spectrometric analysis**

Similar to those in the IP assay, cellular protein extracts from HCC cells were incubated with anti-Flag-TMEM147, followed by incubation with protein A/G agarose beads. The recovered proteins bound with Flag-TMEM147 or IgG were resolved via gel electrophoresis. The bands specifically bound to Flag-TMEM147 were excised, and proteomics screening was performed via mass spectrometric analysis on a MALDI-TOF-MS instrument (Bruker Daltonics).

**Chromatin immunoprecipitation (ChIP)**

ChIP assay was performed using a ChIP Kit (Beyotime, Shanghai, China). Briefly, cells were treated with 1% formalin solution for 10 min and quenched with glycine for 5 min at room temperature to generate DNA–protein cross-links. Cell lysates were sonicated to produce chromatin fragments of 200–1000 bp and immunoprecipitated with anti-DHCR7, anti-STAT2, or IgG antibodies. The immunoprecipitated DNAs was analyzed using qRT-PCR. Information on the primers and antibodies used is provided in Additional File 1: Table S2.

**Dual‑luciferase reporter assay**

The purpose of the dual-luciferase reporter gene assay was to analyze the transcriptional regulation of transcription factors on target genes. The full-length promoter of *DHCR7* carrying mutant or wild-type sequences was cloned into pGLO4.10 vectors (Promega, Madison, WI, USA) and co-transfected with a STAT2 overexpression vector or mock vector into Huh7 cells, using Lipofectamine TM 2000 (Invitrogen, CA, USA). After 48 h of culture, firefly and Renilla luciferase activities were measured using a dual-luciferase reporter gene assay system (Beyotime, Shanghai, China) in accordance with the manufacturer’s protocols.

**Enzyme-linked immunosorbent assay (ELISA) assay**

Cholesteryl Esters’ (CEs) concentrations were measured using a Cholesterol/Cholesterol Ester Quantification Kit (ab65359, Abcam). 27HC concentrations were measured with a Human 27-Hydroxycholesterol ELISA Kit (EH4025, FineTest). Lipid content was measured using a lipid ELISA Kit (SBJ-M0736-96T, GoldenRain/sbj). ELISA was performed according to the manufacturer’s instructions. All experiments were performed in triplicate.

**Glutathione (GSH) Assay**

A reduced glutathione (GSH) assay kit (A006-2-1, Nanjing Jiancheng Bioengineering Institute) was used to measure intracellular GSH levels. Cells (3 × 10^5^) were seeded into a 6-well plate and treated with erastin or RSL3 for 24 h. The cells were harvested, and 0.3 mL PBS was added to the homogenization medium. A 100 μL supernatant was removed for GSH determination. Simultaneously, a standard GSH concentration curve was also generated. The plate was incubated for 5 min after mixing the sample and reagents, and absorbance was measured at 405 nm. The exact GSH concentration in the different cell lines was then calculated based on the GSH standard curve, following the manufacturer’s instructions.

**Mitochondrial iron,** **malondialdehyde, and membrane potential assays**

The mitochondria were isolated using a mitochondrial isolation kit (Thermo Fisher Scientific). Ferrous iron levels in cells or mitochondria were measured using an iron assay kit (Sigma-Aldrich). Lipid peroxidation was assessed in HCC cell lysates by measuring the concentration of malondialdehyde (MDA), an end product of lipid peroxidation, using a lipid peroxidation assay kit (Abcam, Cambridge, MA, USA). Mitochondrial membrane potential (MMP) was measured by staining the cells with 200 nM tetramethylrhodamine ethyl ester (TMRE, Thermo Fisher Scientific) for 20 min. The mean fluorescence intensity of each group was normalized to that of the control group.

**Reactive oxygen species (ROS) assay**

Intracellular ROS generation was detected by reactive oxygen species assay kit (Beyotime Biotechnology, Haimen, China). Before the experiment, 1×10^4^ cells per well were seeded in a 96-well black plate and incubated for 24 h. Following series of indicated experimental treatments, cells were loaded with 10 μM fluorescent probe 2′,7′-dichlorofluorescein diacetate (DCFH-DA) in serum-free DMEM medium for 20 min at 37 ℃. The cells were then washed thrice with serum-free medium. DCF fluorescence intensity was detected using a Tecan Infinite 200 PRO microplate reader to quantify the ROS levels.

**Transmission electron microscopy (TEM)**

TEM was used for ultrastructural analysis of mitochondria. HCC cells were fixed with 2.5% glutaraldehyde in 0.1 M PBS (pH 7.4) at 4 °C for 2.5 h, washed three times with 0.1 M PBS, and post-fixed in 1% OsO_4_ for 2 h at 4 °C. The samples were dehydrated using an ethanol gradient and embedded in Spurr’s resin. Ultrathin sections were collected, stained with uranyl acetate or lead citrate, and examined under a JEOL 1200EX transmission electron microscope.

**Flow cytometric analysis**

PMA-stimulated THP-1 cells that had been co-cultured with HCC cell supernatants for 24 h were stained with PE-conjugated mouse anti-human CD86 (560957, BD Biosciences) or CD206 (555954, BD Biosciences) antibodies according to the manufacturer's instructions. The cells were then fixed and permeabilized using a fixation and permeabilization solution (554722; BD Biosciences). After washing with BD Perm/Wash buffer (554723; BD Biosciences), the cells were stained with an FITC-conjugated anti-human CD68 antibody (562117; BD Biosciences). After 30 min, the cells were washed thrice with PBS and resuspended in 1 mL of FACS buffer for flow cytometry analysis.

**Oxygen consumption rate (OCR) determination**

Cells (2 × 10^4^ cells/well) were seeded in XFe24 seahorse cell culture microplates (Seahorse Bioscience) for 24 h, and the Xfe24 sensor cartridges were hydrated overnight. The cells were treated with a cystine-free medium for 8 h, then the cell medium was replaced with basic seahorse DMEM supplemented with glucose (10 mM), sodium pyruvate (1 mM), and glutamine (2 mM). Subsequently, the cell culture microplate was kept in the CO_2_-free incubator for 1 h, and OCR was measured in real-time with the sequential injection of oligomycin (1.5 μM), carbonyl cyanide chlorophenylhydrazone (CCCP) (2.0 μM), and Antimycin A/Retenone (0.5 μM) by the Seahorse Xfe 24 Bioanalyzer (Seahorse Bioscience).

**Animal model**

Female BALB/c athymic nude mice (4–6 weeks old) were obtained from Beijing Vital River Laboratory Animal Technology Co., Ltd. Mice were housed under specific pathogen-free conditions and raised following institutional guidelines for animal care.

All experimental protocols involving animals were approved by the Animal Ethics Committee of Harbin Medical University, China.

The subcutaneous xenograft model was established by injecting 2 × 10^6^ HCC cells in 200 μL PBS into the flanks of mice. Tumor volume was calculated at the 6^th^ week when all mice were euthanized. Subcutaneous xenograft tumors were cut into 1 mm^3^ cubes and transplanted into the livers (left lobes) of mice to establish an orthotopic tumor model. Tumor size was assessed weekly with NIGHTOWL LB983 system (Berthold Technologies, Wildbad, Germany).

The pulmonary metastasis model was established as follows: 4 × 10^6^ cells suspended in 0.15 mL PBS were injected into the tail vein of each mouse. After 6 weeks, the mice were euthanized, and their lungs were extracted.

All animals were euthanized to collect tumor tissues at the 6th week, and their livers were resected for IHC staining and ELISA.

**Statistical analysis**

Differences between groups were calculated using Student’s *t*-test, the chi-square test, or the Fisher exact test. The probability of differences in survival was ascertained using the Kaplan–Meier method with a log-rank test for significance. Analyses were performed with SPSS version 23.0 and Graphpad Prism 7.0 statistical analysis software. Representative data are shown as mean ± SD. *P* < 0.05 was considered statistically significant.

**Results**

**High expression level of TMEM147 protein is associated with poor survival in HCC.**

To elucidate the relationship between TMEM147 and HCC progression, we first searched the GEO database and mined TCGA transcriptome datasets to compare the levels of TMEM147 between HCC tissues and normal tissues on the GAPIA website and found that *TMEM147* mRNA was significantly upregulated in HCC tissues (Fig. 1a and 1b).

We examined *TMEM147* expression in 72 HCC tissues and 28 non-tumor liver tissues, and found that the TMEM147 protein staining rate in HCC tissues was 83.3% (60/72) compared to 10.7% (3/28) in normal liver tissues (Fig. 1c), and the protein expression level of TMEM147 in the HCC cohort was significantly correlated with patient survival (Fig. 1I). Detailed clinicopathological characteristics are shown in additional file 1: Table S1. Likewise, qRT-PCR in 24 pairs of HCC and peritumoral tissues revealed that the relative mRNA expression of *TMEM147* was significantly upregulated in HCC tissues compared to that in adjacent non-tumorous tissues (Fig. 1d). Accordingly, Western blot analysis was conducted on 24 pairs of HCC tissues, and the results revealed that TMEM147 protein levels were higher in HCC tissues than in non-malignant samples (Fig. 1f and S1a). In agreement with these results, the mRNA and protein expression levels of TMEM147 in HCC cell lines were also significantly higher than those in normal liver cells (Fig. 1e and 1g).

Moreover, Kaplan–Meier survival curve analysis of TCGA datasets demonstrated that HCC patients with increased TMEM147 protein expression had poorer overall five-year survival (Fig. 1h).

**TMEM147 boosts tumor cell proliferation and metastasis**

To investigate the effect of TMEM147 on HCC, we constructed lentivirus vectors to upregulate and silence TMEM147 in Huh7 and HepG2 cells; western blotting and qRT-PCR were used to confirm the overexpression and knockdown efficiency of TMEM147 (Fig. S2a and S2b). According to growth curve analysis and colony formation assay, deletion of *TMEM147* significantly decreased HCC cell proliferation, whereas TMEM147 overexpression significantly increased HCC cell proliferation (Fig. 2a and 2b).

Next, we sought to determine the role of TMEM147 in HCC metastases, migration, for which invasion assays by Transwell and wound healing assays *in vitro* were performed. The results showed that overexpression of *TMEM147* promoted the migration and invasion of Huh7 and HepG2 cells (Fig. 2c and S2c), whereas *TMEM147* knockdown exerted the opposite effect (Fig. 2d and S2d). Collectively, these results indicate that elevated TMEM147 protein level in HCC boosts tumor cell proliferation and metastases.

To evaluate the tumorigenic function of TMEM147 *in vivo*, a subcutaneous xenograft tumor model was established. Consistent with our *in vitro* data, the tumor volume and weight in mice implanted with Huh7-TMEM147 cells were markedly higher than those in the control group; and those in the HepG2-shTMEM147 mice were drastically lower than those in the control group (Fig. 2e). To determine whether TMEM147 affects HCC metastasis *in vivo*, stably transfected cells (Huh7-TMEM147 and controls) were injected into the tail vein to establish a lung metastasis model. Six weeks after the intervention, the number and volume of lung metastases decreased in the *TMEM147* silencing group, but increased in the *TMEM147* overexpression group. (Fig. 2f). In summary, these results provided further evidence that TMEM147 is involved in the exacerbation of HCC growth and metastasis.

**TMEM147 facilitates 27HC expression by activating DHCR7 to accelerate the growth and metastasis of HCC**

To identify the target genes regulated by TMEM147, we knocked down *TMEM147* in Huh7 cells. RNA-seq analysis revealed that the transcriptome profiles of *TMEM147*-silenced cells and the control cells were dissimilar (Fig. 3a). Gene ontology (GO) analysis showed that the differentially expressed genes were significantly enriched in cholesterol metabolism pathways (Fig. 3b). Several key enzymes in this pathway, including methylsterol monooxygenase 1 (MSMO1), emopamil binding protein (EBP), hydroxy-3-methylglutaryl-CoA synthase 1 (HMGCS1), type 2 isopentenyl diphosphate isomerase (IDI2), DHCR7, farnesyl-diphosphate farnesyltransferase (FDFT1), and squalene epoxidase (SQLE), were further assessed. DHCR7 was identified as the most differentially expressed enzyme (Fig. 3c and S3a), implying that the expression of DHCR7 may be modulated by *TMEM147*.

To confirm the role of TMEM147 in the regulation of DHCR7, the expression of DHCR7 in HCC cells was detected after *TMEM147* was knocked down or overexpressed. TMEM147 overexpression increased *DHCR7* transcription and protein levels, whereas DHCR7 levels were significantly reduced in the absence of TMEM147 (Fig. 3d and 3e).

CEs, the intracellular storage forms of excess cholesterol, are of central importance to cholesterol homeostasis; their formation is a measure of the availability of cellular free cholesterol, and CE levels are consistent with sterol reductase activity.^[31]^ ELISA was employed to quantify the CEs in cell lysates and subcutaneous tumors from mice. The results indicate that the total levels of CEs are greatly reduced after *TMEM147* knockdown and are significantly upregulated after TMEM147 overexpression (Fig. 3f). A study showed that in breast cancer, cholesterol mediates the metastatic effects via its oxysterol metabolite, 27-hydroxycholesterol.^[28]^ Therefore, we next detected the levels of 27HC in cells and the cell culture medium using ELISA. As anticipated, the levels of 27HC were consistent with those of CEs and TMEM147 (Fig. 3g).

CYP27A1 is the enzyme responsible for the rate-limiting step in 27HC biosynthesis, and we found that the protein and mRNA levels of CYP27A1 were closely correlated with TMEM147 levels (Fig. 3h and S3b). To explore the role of DHCR7 in the tumor-promoting function of TMEM147, the effect of DHCR7 knockdown on the tumorigenic potential of TMEM147, RNA lentivirus stably transfected into Huh7 and HepG2 cells was examined (Fig. S3c and S3d). Downregulation of DHCR7 expression markedly suppressed the proliferative, migratory, and invasive capabilities of HCC cells, whereas the addition of exogenous 27HC partially restored the inhibitory effect of DHCR7 silencing. The function of 27HC was impeded by the CYP27A1 inhibitor, GW297X (Fig. 3i, S3e, 3j, and S3f).

A xenograft model showed that *DHCR7* knockdown inhibited the growth of subcutaneous tumors, whereas 27HC supplementation reversed the inhibitory effect of DHCR7 downregulation on tumor growth. Additionally, *DHCR7* knockdown decreases lung metastasis in a mouse model. However, 27HC supplementation neutralized the inhibition of lung metastasis by *DHCR7* knockdown, whereas the injection of the CYP27A1 inhibitor GW297X in cells overexpressing TMEM147 restrained tumor progression and metastasis, and supplementation with 27HC offset the effect of GW297X (Fig. S3g and S3h). Collectively, the *in vitro* and *in vivo* results suggest that DHCR7 is involved in the oncogenic effect of TMEM147 in HCC and that the function of DHCR7 depends on increased 27HC levels.

**TMEM147-mediated promotion of DHCR7 expression is dependent on the transcription factor STAT2**

In Huh7 and HepG2 cell lines, protein binding to TMEM147 was analyzed by mass spectrometry using Co-IP. It was found that the target gene *STAT2,* which encodes a transcription factor was closely bound to TMEM147 in both cell lines (Fig. 4a). The interaction between TMEM147 and STAT2 was further confirmed by Co-IP. IP assays confirmed that STAT2 existed in complexes and was precipitated with an antibody against Flag-TMEM147, unlike with the control IgG (Fig. 4b, upper panel). The binding of endogenous STAT2 to TMEM147 was validated by an IP assay using an antibody against STAT2 (Fig. 4b, lower panel). Using JASPAR and UCSC database analyses, we found that the DHCR7 promoter contained putative binding sites for the transcription factor STAT2 (Fig. 4c). We speculated that TMEM147 might regulate DHCR7 expression through the transcription factor STAT2.

As shown in Fig. 3, TMEM147 altered the mRNA and protein expression of DHCR7. Western blotting revealed that STAT2 protein expression levels did not significantly correlate with those of TMEM147 in HCC cells. However, p-STAT2 (STAT2 phosphorylated at Tyr690) levels changed upon TMEM147 treatment (Fig. 4d). Consistent with this observation, ChIP assay showed that the STAT2-bound *DHCR7* promoter was enriched in TMEM147 overexpressed cells but diminished in TMEM147-knocked down cells (Fig. 4e). The luciferase reporter assay showed that STAT2 overexpression significantly increased the luciferase activity of reporters containing wild-type binding sites compared to that of NC-vector cells (Fig. 4f). However, no significant change in luciferase activity was observed upon binding of STAT2 to the mutant *DHCR7* promoter (Fig. 4f). To determine whether STAT2 influenced DHCR7 expression, we transfected STAT2 into HCC cells. We found that STAT2 significantly promoted the expression levels of DHCR7 in HCC cells; in contrast, elimination of STAT2 significantly decreased DHCR7 expression (Fig. 4g). When TMEM147 was overexpressed, si-STAT2 inhibited DHCR7 expression, which indicating that the TMEM147-mediated function of DHCR7 depended on STAT2 (Fig. 4h). A moderate correlation between TMEM147 and STAT2, and DHCR7 and STAT2 was detected in human HCC (Fig. 4i), suggesting that TMEM147 promotes the phosphorylation of STAT2, enhances its transcriptional activity, and then increases the expression of DHCR7. To assess the effect of STAT2 on HCC, we transfected si-STAT2 into HCC cells and found si-STAT2 significantly suppressed HCC cell proliferation, invasion, and migration (Fig. 4j and 4k).

**TMEM147 induces ferroptosis by promoting activation of the 27HC/GPX4 pathway in HCC cells**

Previous studies have shown that chronic exposure to 27HC increases lipid accumulation in cells and enhances the resistance of breast cancer cells to ferroptosis, whereas GPX4 inhibition reverses this effect.^[20]^ To explore the effects of TMEM147 and 27HC on ferroptosis, we measured the levels of GSH, which plays an essential role in the repair of oxidative damage. As shown in Fig. 5a, erastin and RSL3 significantly decreased GSH levels in HCC cells, and TMEM147 overexpression rescued the decreased GSH levels caused by erastin and RSL3. When GW297X was added, the GSH value decreased significantly. Correspondingly, ferrous ion (Fe^2+^) and MDA levels were reduced when TMEM147 was overexpressed but increased in cells with TMEM147 overexpression subjected to GW297X treatment (Fig. S5a and S5b). MMP increased over time, to a maximum at approximately 8 h after erastin treatment (Fig. 5b); however, MMP was overwhelmingly suppressed by TMEM147, and completely rescued by GW297X. Correspondingly, as shown in Fig. 5c, TMEM147 reduced ROS production via 27HC, whereas ROS generation was significantly enhanced in GW297X-treated cells.

TEM analysis further revealed that erastin and RSL3 treated HCC cells contained shrunken mitochondria with elevated membrane density, a typical morphological feature of ferroptosis. TMEM147 overexpression protected cells from ferroptosis, whereas treatment with GW297X aggravated ferroptosis. This indicates that TMEM147 confers ferroptosis resistance by promoting 27HC. (Fig. 5d)

In parallel with our observation *in* *vitro*, tumor size and weight in the erastin group were significantly increased by TMEM147 overexpression, whereas GW297X pretreatment markedly reversed this TMEM147‐mediated oncogenic effect (Fig. 5e). Tissues from the control and GW297X injected tumors, exhibited increased levels of 4-hydroxy-2-noneal (4HNE, a biomarker of lipid peroxidation), whereas TMEM147 overexpression reduced the levels of 4HNE, as determined via IHC (Fig. 5f). These *in* *vivo* results further support the hypothesis that the resistance to ferroptosis induced by TMEM147 is mainly dependent on 27HC.

To further elucidate the mechanism of ferroptosis triggered by TMEM147, the expression of the iron metabolism-related proteins GPX4, HMOX1, SLC7A11, NCOA4, NRF2, and CP was analyzed. GPX4 was markedly altered by TMEM147 and GW297X (Fig. 5g and S5c). The expression of GPX4 in HCC cells was assessed by qRT-PCR and western blot analysis, and it was found that GPX4 expression was downregulated upon the knockdown of TMEM147. Indeed, we demonstrated that the TMEM147-dependent GPX4 downregulation was dramatically rescued by the addition of exogenous 27HC. (Fig. 5h and 5i). In summary, these results suggested that TMEM147 promotes GPX4 expression via 27HC to reduce the resistance of HCC cells to ferroptosis.

**HCC cell-derived** **27HC enhances lipid metabolism in macrophages and drives M2 polarization**

Previous studies have shown that pretreatment of macrophages with 27HC can inhibit T cell function.^[30]^ However, it remains unclear whether tumor-associated macrophages, which are important immune cells in the TME, are also regulated by 27HC. To further substantiate this hypothesis, the following experiments were conducted.

THP-1 induced macrophages were incubated with CM extracted from HCC cells (Fig. 6a), and the expression of TAM markers arginase 1 (ARG1) and CD206 was further analyzed. CM from TMEM147 overexpression cells or the addition of exogenous 27HC, but not the control medium, increased the expression of the M2 markers ARG1 and CD206, whereas GW297X deterred this conversion (Fig. 6b and S6a). These results suggest that endogenous 27HC mediates M2 macrophage polarization. The results of cell immunofluorescence and flow cytometry further confirmed that tumor-derived 27HC was responsible for cancer CM-induced M2 macrophage polarization (Fig. 6c and 6d).

In animal studies, IHC staining for the M2 marker ARG1 showed that the accumulation of M2 macrophages was greater in macrophages cultured with the CM obtained from TMEM147 overexpression cells or with exogenous 27HC (Fig. 6e).

Pan Su *et.al*.^[32]^ reported that the reprogramming of fatty acid oxidative metabolism plays a crucial role in M2 polarization of TAMs. Macrophages from both human and murine tumor tissues are enriched with lipids owing to increased lipid uptake by macrophages. In our study, as expected, compared with control, the macrophages cultured with CM extracted from TMEM147 overexpression cells or added exogenous 27HC were enriched with more lipids (Fig. 6f), genes involved in fatty acid β-oxidation including FAO rate-limiting enzymes CPT1A and PPARγ, were significantly increased (Fig. 6b and S6a). In metabolic assay experiments, TAMs co-cultured with TMEM147 overexpression cells and exogenous 27HC supplementation displayed enhanced mitochondrial OCR and markedly increased spare respiratory capacity (SRC), in contrast to TMEM147 knockdown cells co-cultured with GW297X-treated macrophages (Fig. 6g and 6h). Thus, we validated that 27HC promotes the accumulation of lipids and fatty acid-oxidation in TAMs, which are crucial for M2 macrophage polarization.

In summary, we highlighted the importance of HCC cell-derived 27HC in enhancing the lipid metabolism of macrophages and promoting M2 polarization.

**27HC-induced polarized macrophages potentiate migration of HCC cells**

As cancer cell-derived 27HC can promote the M2 polarization of TAMs, we hypothesized that TAMs with increased M2 polarization could further promote the migration of HCC cells. CM-induced polarized macrophages were co-cultured with HCC cells (Fig. 7a), and the invasion and migration of HCC cells in each group were consistent with M2 polarization of macrophages (Fig. 7b and 7c). *In* *vivo*, we injected co-cultured cells through the tail vein of nude mice and monitored lung metastatic nodules (Fig. 7d), which showed that polarized macrophages induced by 27HC could significantly exacerbate the metastasis of HCC cells.

**Discussion**

HCC is one of the most common life-threatening form of liver cancer, with a high incidence and mortality worldwide. In the present study, we identified *TMEM147* as a potent oncogene responsible for HCC proliferation and metastasis. *TMEM147* can enhance the transcription of DHCR7 (the key enzyme for cholesterol synthesis) through the transcription factor STAT2 and alter cholesterol homeostasis in HCC. Consequently, both the intracellular as well as extracellular levels of 27HC, a primary metabolite of cholesterol, increase. We found that 1) 27HC promoted the proliferation, migration and invasion of HCC cells *in vivo* and *in vitro*; 2) 27HC inhibited the ferroptosis in tumor cells; and 3) 27HC expelled out of the cell to promote polarization of the M2 macrophages, which helps HCC cell invasion and metastasis.

In this study, we selected TMEM147 as a promising research target through GEO and TCGA data analysis of patients with HCC. Accordingly, our data showed that high TMEM147 expression was linked to worse OS and DFS in patients with HCC. We confirmed that knockdown of *TMEM147* expression arrests HCC cell proliferation and reduces their migration and invasion potential, both *in vitro* and *in vivo*. Conversely, we found that DHCR7 expression was regulated by TMEM147. A previous study has reported a correlation between TMEM147 and DHCR7 expression in HeLa cells; however, the underlying mechanism remains unclear. Our results were similar to those in HeLa cells, and we explored the potential underlying mechanisms. DHCR7 has crucial functions in several malignant diseases such as gastric cancer^[13]^ and colorectal cancer.^[14]^ In this study, we found that the positive effect of TMEM147 on the proliferation, invasion, and migration of HCC cells mainly relies on DHCR7, which is related to the DHCR7-modulated cholesterol ester metabolism.

Ota *et al*.^[7]^ found that TMEM147 has a role in NF-κB activation in human primary chondrocytes. There are multiple phosphorylation sites on NF-κB molecules. For instance, phosphorylation of p65 S276 residue is important for DNA binding, and TMEM147 can phosphorylate p65 S529. At the molecular level, our investigation revealed that TMEM147 induced the phosphorylation of the transcription factor STAT2 and promoted the transcriptional activity of STAT2, which binds to the transcription promoter regions of DHCR7 and further accelerates the transcription of DHCR7. DHCR7 promotes accumulation of cholesteryl esters in cells and increases 27HC levels in the TME.

Our research validated that 27HC is an important lipid metabolite regulated by TMEME147. 27HC plays a contradictory role in the progression of various cancers; for example, 27HC can increase ER-dependent growth and LXR-dependent metastasis in mouse models of breast cancer ^[30, 33, 34]^; 27HC promotes lung cancer cell proliferation via ERβ and PI3K-Akt signaling,^[35]^ whereas in colorectal cancer, increased production of 27HC inhibits cancer cell survival and infiltration.^[36]^ Therefore, it is unclear whether 27HC promotes or suppresses malignant tumors. This study revealed that 27HC promotes HCC growth. Ferroptosis is a newly discovered mode of cell death, and multiple signaling pathways dictate the susceptibility of cells to ferroptosis. Unrestrained lipid peroxidation is the hallmark of ferroptosis. The cyst(e)ine-GSH-GPX4 axis is considered the main ferroptosis-regulating system in mammals.^[37]^ FSP1 is another prominent ferroptosis suppressor.^[38]^ The role of the E-cadherin-NF2-Hippo-YAP pathway in ferroptosis sensitivity has important implications.^[39]^ Hypoxia can drive sensitivity to ferroptosis; hence, the HIF2α-HILPDA pathway drives sensitization to ferroptosis.^[40]^ However, upstream regulatory pathways involved in ferroptosis remain unclear. Additionally, a study reported that 27HC affects the pathogenesis of breast cancer by selecting cells that are resistant to ferroptosis.^[20]^ A previous study has reported that ZMYND8 is a master regulator of 27HC, which promotes the tumorigenicity of breast cancer stem cells. Our study demonstrated that TMEM147 regulates 27HC production mainly by activating DHCR7. Furthermore, TMEM147 confers resistance to ferroptosis which is primarily dependent on 27HC. To further address the specific mechanism by which 27HC inhibits ferroptosis, we detected key proteins involved in several ferroptotic pathways when TMEM147 was altered. Ultimately, GPX4 was identified as a downstream target of 27HC, which is consistent with previous studies on other tumors.

Recent studies have revealed that 27HC affects cellular functions in the TME. 27HC increases γδT cells and polymorphonuclear neutrophils but decreases CD8+ T cells at distal metastatic sites in breast cancer.^[28]^ 27HC also facilitates osteoclast differentiation by activating the STAT3 signaling pathway, thus providing an appropriate bone microenvironment for the colonization of lung cancer cells.^[29]^ Additionally, evidence suggests that 27HC is critical for promoting the pro-tumorigenic properties of myeloid cells and the abundance and plasticity of macrophages within the tumor microenvironment.^[30]^ Further investigation demonstrated that 27HC might be released into the TME and contribute in the FAO metabolic reprogramming of TAMs. To date, no studies have explored whether 27HC affects TAM polarization. Macrophages are the most abundant mesenchymal cells in HCC, and macrophage polarization plays a significant regulatory role in HCC progression.^[41, 42]^ We speculated that the release of 27HC induced by TMEM147 may further orchestrate the polarization of TAMs; therefore, we altered the expression of TMEM147 and 27HC, and cultured macrophages with CM from the treated HCC cells to simulate the TME *in vivo*. Finally, consistent with our hypothesis, HCC cell-derived 27HC significantly increased the M2 polarization, which further confirmed the potential mechanism of TMEM147 in HCC cells in that it influenced TAM M2 polarization by regulating the release of 27HC. Recent research has reported that fatty acid oxidative metabolic reprogramming plays a crucial role in M2 polarization of TAMs.^[32]^ Therefore, we detected key proteins in the FAO pathway in macrophages and found that the M2 polarization induced by 27HC was indeed caused by FAO metabolic reprogramming. We further co-cultured macrophages with HCC cells and confirmed that M2 polarized macrophage aggravated HCC invasion and metastasis in a positive feedback manner.

Our results confirm that TMEM147-mediated metabolic reprogramming and the inflammatory microenvironment play vital roles in HCC progression. Future studies should further screen or develop novel compounds that interrupt the TMEM147/STAT2/DHCR7/27HC pathway, which may be a potential strategy for therapy and HCC immunotherapy.

**Conclusion**

We demonstrated, for the first time, that TMEM147 is a critical regulator of HCC cell proliferation and metastasis. TMEM147 binds to the transcription factor STAT2 and increases its phosphorylation; phosphorylated STAT2 directly bind to the predicted promoter region of the *DHCR7* gene, further promoting the transcription of DHCR7. This alters cholesterol homeostasis in HCC, increasing 27HC levels, which leads to increased GPX4 and protects cells from ferroptosis in HCC cells; furthermore, it can also be discharged into the TME, activating the PPAR/CPTA1 pathway, causing lipid accumulation in macrophages, resulting in TAM metabolic reprogramming, and consequently promoting M2 polarization. Collectively, these results provide a foundation for understanding the mechanisms underlying HCC progression, and may contribute to the identification of new biomarkers and therapeutic targets for HCC. In the present study, we identified 27HC as a target regulating the proliferation, invasion, and metastasis of HCC. We also demonstrated that 27HC induces ferroptosis resistance. Nevertheless, the possibility that 27HC could accelerate the progression of HCC via additional death mechanisms cannot be excluded; thus, further research on this aspect is necessary.

**Abbreviations**

27HC: 27-hydroxycholesterol; CCK-8: Cell counting Kit-8; CEs: cholesteryl esters; ChIP: chromatin immunoprecipitation; DCF: 2′,7′-dichlorofluorescein; DCFH-DA: DCF diacetate; DHCR7: 7-dehydrocholesterol reductase; DHCR7: 7-dehydrocholesterol reductase; EBP: emopamil binding protein; ELISA: enzyme-linked immunosorbent assay; ER: endoplasmic reticulum; FAO: fatty acid oxidation; FDFT1: farnesyl-diphosphate farnesyltransferase; GO: Gene ontology; GPX4: glutathione peroxidase 4; GSH: glutathione; HCC: hepatocellular carcinoma; HMGCS1: hydroxy-3-methylglutaryl-CoA synthase 1; IDI2: type 2 isopentenyl diphosphate isomerase; IHC: immunohistochemistry; IP: immunoprecipitation; MDA: malondialdehyde; MMP: mitochondrial membrane potential; MSMO1; methylsterol monooxygenase 1; OCR: oxygen consumption rate; PBS: phosphate buffered saline; PMA: phorbol myristate acetate; qRT-PCR: quantitative real-time polymerase chain reaction; ROS: reactive oxygen species; SQLE: squalene epoxidase; STAT2: signal transducer and activator of transcription 2; TAM: Tumor-associated macrophages; TEM: transmission electron microscopy; TCGA: The Cancer Genome Atlas; TME: tumor microenvironment; TMEM147: transmembrane protein 147.

**Declarations**

**Ethics approval and consent to participate**

The present study was approved by the Ethics Committee of the First affiliated Hospital of Harbin Medical University, and was performed according to the Declaration of Helsinki. All patients recruited into the study signed written informed consent forms.

**Consent for publication**

**Availability of data and materials**

The datasets supporting the conclusions of this article are included within the article and its additional files.

**Competing interests**

The authors declare no competing interests.

**Funding**

This work was supported by Chen Xiao-Ping Foundation For The Development Of Science And Technology Of Hubei Province (grant CXPJJH12-2020023)，the Excellent Young Medical Talents Training Fund of the First Affiliated Hospital of Harbin Medical University (grant 2021Y06), Heilongjiang Postdoctoral Scientific Research Developmental Fund (grant LBH-Q18089), The University Nursing Program for Young Scholars with Creative Talents in Heilongjiang Province (grant UNPYSCT-2018070), Chen Xiao-Ping Foundation For The Development Of Science And Technology Of Hubei Province (grant CXPJJH12-2021029).

**Author contributions**

JH, HP, and JS performed the experiments, analyzed the data, finalized the figures, and drafted the manuscript. JW, QX, SK, SL, and ZL performed the experiments. YH, ZF, BY, and BQ assisted with the data analysis and figure design. HP, HJ, and WD designed and supervised the study. All the authors contributed to and approved the final version of the manuscript.

**Acknowledgements**

Not applicable

Reference

[1] Hepatocellular carcinoma [J]. Journal, 2021, 7(Issue): 7.

[2] Dettmer U, P Kuhn, C Abou-Ajram, S Lichtenthaler, M Krüger, E Kremmer, C Haass and C Haffner. Transmembrane protein 147 (TMEM147) is a novel component of the Nicalin-NOMO protein complex [J]. Journal, 2010, 285(Issue): 26174-81.

[3] Chen S, P Novick and S Ferro-Novick. ER structure and function [J]. Journal, 2013, 25(Issue): 428-33.

[4] McGilvray P, S Anghel, A Sundaram, F Zhong, M Trnka, J Fuller, H Hu, A Burlingame and R Keenan. An ER translocon for multi-pass membrane protein biogenesis [J]. Journal, 2020, 9(Issue):

[5] Rosemond E, M Rossi, S McMillin, M Scarselli, J Donaldson and J Wess. Regulation of M₃ muscarinic receptor expression and function by transmembrane protein 147 [J]. Journal, 2011, 79(Issue): 251-61.

[6] Wu T, N Han, C Zhao, X Huang, P Su and X Li. The long non-sacoding RNA TMEM147-AS1/miR-133b/ZNF587 axis regulates the Warburg effect and promotes prostatic carcinoma invasion and proliferation [J]. Journal, 2022, 24(Issue): e3453.

[7] Ota M, Y Tanaka, I Nakagawa, J J Jiang, Y Arima, D Kamimura, T Onodera, N Iwasaki and M Murakami. Role of Chondrocytes in the Development of Rheumatoid Arthritis Via Transmembrane Protein 147-Mediated NF-kappaB Activation [J]. Journal, 2020, 72(Issue): 931-942.

[8] Feng Y, Y Li, L Li, X Wang and Z Chen. Identification of specific modules and significant genes associated with colon cancer by weighted gene coexpression network analysis [J]. Journal, 2019, 20(Issue): 693-700.

[9] Prabhu A V, W Luu, D Li, L J Sharpe and A J Brown. DHCR7: A vital enzyme switch between cholesterol and vitamin D production [J]. Journal, 2016, 64(Issue): 138-151.

[10] Zou J, S Liu, J Long and B Yan. High DHCR7 Expression Predicts Poor Prognosis for Cervical Cancer [J]. Journal, 2022, 2022(Issue): 8383885.

[11] Li Y, Y Zhou, M Huang, Z Wang, D Liu, J Liu, X Fu, S Yang, S Shan, L Yang, Y Guo, P Ren, P Chen, G Zeng, Y Guo, X Wang, M DiSanto and X Zhang. DHCR7 promotes tumorigenesis via activating PI3K/AKT/mTOR signalling pathway in bladder cancer [J]. Journal, 2023, 102(Issue): 110553.

[12] Ong J, G Cuellar-Partida, Y Lu, P Fasching, A Hein, S Burghaus, M Beckmann, D Lambrechts, E Van Nieuwenhuysen, I Vergote, A Vanderstichele, J Anne Doherty, M Anne Rossing, J Chang-Claude, U Eilber, A Rudolph, S Wang-Gohrke, M Goodman, N Bogdanova, T Dörk, M Dürst, P Hillemanns, I Runnebaum, N Antonenkova, R Butzow, A Leminen, H Nevanlinna, L Pelttari, R Edwards, J Kelley, F Modugno, K Moysich, R Ness, R Cannioto, E Høgdall, C Høgdall, A Jensen, G Giles, F Bruinsma, S Kjaer, M Hildebrandt, D Liang, K Lu, X Wu, M Bisogna, F Dao, D Levine, D Cramer, K Terry, S Tworoger, M Stampfer, S Missmer, L Bjorge, H Salvesen, R Kopperud, K Bischof, K Aben, L Kiemeney, L Massuger, A Brooks-Wilson, S Olson, V McGuire, J Rothstein, W Sieh, A Whittemore, L Cook, N Le, C Gilks, J Gronwald, A Jakubowska, J Lubiński, T Kluz, H Song, J Tyrer, N Wentzensen, L Brinton, B Trabert, J Lissowska, J McLaughlin, S Narod, C Phelan, H Anton-Culver, A Ziogas, D Eccles, I Campbell, S Gayther, A Gentry-Maharaj, U Menon, S Ramus, A Wu, A Dansonka-Mieszkowska, J Kupryjanczyk, A Timorek, L Szafron, J Cunningham, B Fridley, S Winham, E Bandera, E Poole, T Morgan, H Risch, E Goode, J Schildkraut, C Pearce, A Berchuck, P Pharoah, G Chenevix-Trench, P Gharahkhani, R Neale, P Webb and S MacGregor. Association of vitamin D levels and risk of ovarian cancer: a Mendelian randomization study [J]. Journal, 2016, 45(Issue): 1619-1630.

[13] Chen Y, W Yan, K Yang, Y Qian, Y Chen, R Wang, J Zhu, Y He, H Wu, G Zhang, T Shi and W Chen. Integrated multi-dimensional analysis highlights DHCR7 mutations involving in cholesterol biosynthesis and contributing therapy of gastric cancer [J]. Journal, 2023, 42(Issue): 36.

[14] Wen J, J Li, X Liang and A Wang. DHCR7Association of Polymorphisms in Vitamin D-Metabolizing Enzymes and with Cancer Susceptibility: A Systematic Review and Meta-Analysis [J]. Journal, 2021, 2021(Issue): 6615001.

[15] Ma H, G Yamamoto, J Xu, X Liu, D Karin, J Kim, L Alexandrov, Y Koyama, T Nishio, C Benner, S Heinz, S Rosenthal, S Liang, M Sun, G Karin, P Zhao, P Brodt, I Mckillop, O Quehenberger, E Dennis, A Saltiel, H Tsukamoto, B Gao, M Karin, D Brenner and T Kisseleva. IL-17 signaling in steatotic hepatocytes and macrophages promotes hepatocellular carcinoma in alcohol-related liver disease [J]. Journal, 2020, 72(Issue): 946-959.

[16] Fitzky B, F Moebius, H Asaoka, H Waage-Baudet, L Xu, G Xu, N Maeda, K Kluckman, S Hiller, H Yu, A Batta, S Shefer, T Chen, G Salen, K Sulik, R Simoni, G Ness, H Glossmann, S Patel and G Tint. 7-Dehydrocholesterol-dependent proteolysis of HMG-CoA reductase suppresses sterol biosynthesis in a mouse model of Smith-Lemli-Opitz/RSH syndrome [J]. Journal, 2001, 108(Issue): 905-15.

[17] Teplyuk N, Y Zhang, Y Lou, J Hawse, M Hassan, V Teplyuk, J Pratap, M Galindo, J Stein, G Stein, J Lian and A van Wijnen. The osteogenic transcription factor runx2 controls genes involved in sterol/steroid metabolism, including CYP11A1 in osteoblasts [J]. Journal, 2009, 23(Issue): 849-61.

[18] Wang J, R Shi, Q Yang, Z Chen, J Wang, Z Gong, S Chen and N Wang. Characterization and potential function of 7-dehydrocholesterol reductase (dhcr7) and lathosterol 5-desaturase (sc5d) in Cynoglossus semilaevis sexual size dimorphism [J]. Journal, 2023, 853(Issue): 147089.

[19] Yang M, X Wu, J Hu, Y Wang, Y Wang, L Zhang, W Huang, X Wang, N Li, L Liao, M Chen, N Xiao, Y Dai, H Liang, W Huang, L Yuan, H Pan, L Li, L Chen, L Liu, L Liang and J Guan. COMMD10 inhibits HIF1α/CP loop to enhance ferroptosis and radiosensitivity by disrupting Cu-Fe balance in hepatocellular carcinoma [J]. Journal, 2022, 76(Issue): 1138-1150.

[20] Liu W, B Chakraborty, R Safi, D Kazmin, C Y Chang and D P McDonnell. Dysregulated cholesterol homeostasis results in resistance to ferroptosis increasing tumorigenicity and metastasis in cancer [J]. Journal, 2021, 12(Issue): 5103.

[21] Lu C S, A L Shiau, B H Su, T S Hsu, C T Wang, Y C Su, M S Tsai, Y H Feng, Y L Tseng, Y T Yen, C L Wu and G S Shieh. Oct4 promotes M2 macrophage polarization through upregulation of macrophage colony-stimulating factor in lung cancer [J]. Journal, 2020, 13(Issue): 62.

[22] Liang X, M Luo, B Shao, J Yang, A Tong, R Wang, Y Liu, R Jun, T Liu, T Yi, X Zhao, Y Wei and X Wei. Phosphatidylserine released from apoptotic cells in tumor induces M2-like macrophage polarization through the PSR-STAT3-JMJD3 axis [J]. Journal, 2022, 42(Issue): 205-222.

[23] Wu J, T Huang, Y Hsieh, Y Wang, C Yen, G Lee, C Yeh, Y Peng, Y Kuo, H Wen, H Lin, C Hsiao, K Wu, H Kung, Y Hsu and C Kuo. Cancer-Derived Succinate Promotes Macrophage Polarization and Cancer Metastasis via Succinate Receptor [J]. Journal, 2020, 77(Issue): 213-227.e5.

[24] Noe J, B Rendon, A Geller, L Conroy, S Morrissey, L Young, R Bruntz, E Kim, A Wise-Mitchell, M Barbosa de Souza Rizzo, E Relich, B Baby, L Johnson, H Affronti, K McMasters, B Clem, M Gentry, J Yan, K Wellen, R Sun and R Mitchell. Lactate supports a metabolic-epigenetic link in macrophage polarization [J]. Journal, 2021, 7(Issue): eabi8602.

[25] Zhao S, Y Mi, B Guan, B Zheng, P Wei, Y Gu, Z Zhang, S Cai, Y Xu, X Li, X He, X Zhong, G Li, Z Chen and D Li. Tumor-derived exosomal miR-934 induces macrophage M2 polarization to promote liver metastasis of colorectal cancer [J]. Journal, 2020, 13(Issue): 156.

[26] Wang F, Y Niu, K Chen, X Yuan, Y Qin, F Zheng, Z Cui, W Lu, Y Wu and D Xia. Extracellular Vesicle-Packaged circATP2B4 Mediates M2 Macrophage Polarization via miR-532-3p/SREBF1 Axis to Promote Epithelial Ovarian Cancer Metastasis [J]. Journal, 2023, 11(Issue): 199-216.

[27] Ubil E, L Caskey, A Holtzhausen, D Hunter, C Story and H Earp. Tumor-secreted Pros1 inhibits macrophage M1 polarization to reduce antitumor immune response [J]. Journal, 2018, 128(Issue): 2356-2369.

[28] Baek A, Y Yu, S He, S Wardell, C Chang, S Kwon, R Pillai, H McDowell, J Thompson, L Dubois, P Sullivan, J Kemper, M Gunn, D McDonnell and E Nelson. The cholesterol metabolite 27 hydroxycholesterol facilitates breast cancer metastasis through its actions on immune cells [J]. Journal, 2017, 8(Issue): 864.

[29] Zhang L, M Liu, J Liu, X Li, M Yang, B Su and Y Lin. 27-Hydroxycholesterol enhanced osteoclastogenesis in lung adenocarcinoma microenvironment [J]. Journal, 2019, 234(Issue): 12692-12700.

[30] Ma L, L Wang, A Nelson, C Han, S He, M Henn, K Menon, J Chen, A Baek, A Vardanyan, S Shahoei, S Park, D Shapiro, S Nanjappa and E Nelson. 27-Hydroxycholesterol acts on myeloid immune cells to induce T cell dysfunction, promoting breast cancer progression [J]. Journal, 2020, 493(Issue): 266-283.

[31] Luo J, H Yang and B Song. Mechanisms and regulation of cholesterol homeostasis [J]. Journal, 2020, 21(Issue): 225-245.

[32] Wu L, X Zhang, L Zheng, H Zhao, G Yan, Q Zhang, Y Zhou, J Lei, J Zhang, J Wang, R Xin, L Jiang, J Peng, Q Chen, S Lam, G Shui, H Miao and Y Li. RIPK3 Orchestrates Fatty Acid Metabolism in Tumor-Associated Macrophages and Hepatocarcinogenesis [J]. Journal, 2020, 8(Issue): 710-721.

[33] Nelson E, S Wardell, J Jasper, S Park, S Suchindran, M Howe, N Carver, R Pillai, P Sullivan, V Sondhi, M Umetani, J Geradts and D McDonnell. 27-Hydroxycholesterol links hypercholesterolemia and breast cancer pathophysiology [J]. Journal, 2013, 342(Issue): 1094-8.

[34] Lu D, C Le Cornet, D Sookthai, T Johnson, R Kaaks and R Fortner. Circulating 27-Hydroxycholesterol and Breast Cancer Risk: Results From the EPIC-Heidelberg Cohort [J]. Journal, 2019, 111(Issue): 365-371.

[35] Hiramitsu S, T Ishikawa, W Lee, T Khan, C Crumbley, N Khwaja, F Zamanian, A Asghari, M Sen, Y Zhang, J Hawse, J Minna and M Umetani. Estrogen Receptor Beta-Mediated Modulation of Lung Cancer Cell Proliferation by 27-Hydroxycholesterol [J]. Journal, 2018, 9(Issue): 470.

[36] Rossin D, I H K Dias, M Solej, I Milic, A R Pitt, N Iaia, L Scoppapietra, A Devitt, M Nano, M Degiuli, M Volante, C Caccia, V Leoni, H R Griffiths, C M Spickett, G Poli and F Biasi. Increased production of 27-hydroxycholesterol in human colorectal cancer advanced stage: Possible contribution to cancer cell survival and infiltration [J]. Journal, 2019, 136(Issue): 35-44.

[37] Yang W, R SriRamaratnam, M Welsch, K Shimada, R Skouta, V Viswanathan, J Cheah, P Clemons, A Shamji, C Clish, L Brown, A Girotti, V Cornish, S Schreiber and B Stockwell. Regulation of ferroptotic cancer cell death by GPX4 [J]. Journal, 2014, 156(Issue): 317-331.

[38] Doll S, F Freitas, R Shah, M Aldrovandi, M da Silva, I Ingold, A Goya Grocin, T Xavier da Silva, E Panzilius, C Scheel, A Mourão, K Buday, M Sato, J Wanninger, T Vignane, V Mohana, M Rehberg, A Flatley, A Schepers, A Kurz, D White, M Sauer, M Sattler, E Tate, W Schmitz, A Schulze, V O'Donnell, B Proneth, G Popowicz, D Pratt, J Angeli and M Conrad. FSP1 is a glutathione-independent ferroptosis suppressor [J]. Journal, 2019, 575(Issue): 693-698.

[39] Wu J, A Minikes, M Gao, H Bian, Y Li, B Stockwell, Z Chen and X Jiang. Intercellular interaction dictates cancer cell ferroptosis via NF2-YAP signalling [J]. Journal, 2019, 572(Issue): 402-406.

[40] Zou Y, M Palte, A Deik, H Li, J Eaton, W Wang, Y Tseng, R Deasy, M Kost-Alimova, V Dančík, E Leshchiner, V Viswanathan, S Signoretti, T Choueiri, J Boehm, B Wagner, J Doench, C Clish, P Clemons and S Schreiber. A GPX4-dependent cancer cell state underlies the clear-cell morphology and confers sensitivity to ferroptosis [J]. Journal, 2019, 10(Issue): 1617.

[41] Zhou C, J Weng, C Liu, S Liu, Z Hu, X Xie, D Gao, Q Zhou, J Sun, R Xu, H Li, Y Shen, Y Yi, Y Shi, X Sheng, Q Dong, M Hung and N Ren. Disruption of SLFN11 Deficiency-Induced CCL2 Signaling and Macrophage M2 Polarization Potentiates Anti-PD-1 Therapy Efficacy in Hepatocellular Carcinoma [J]. Journal, 2023, (Issue):

[42] Wang L, Y Hu, J Zhao, F Huang, S Liang, L Dong, Y Chen, H Yu, J Bai, J Yang, J Fan, L Feng, S Li, H Han and H Qin. Targeted delivery of miR-99b reprograms tumor-associated macrophage phenotype leading to tumor regression [J]. Journal, 2020, 8(Issue):

**Figure legends**

**Fig. 1 TMEM147 was highly expressed in HCC and correlates with poor prognosis in HCC patients.**

(a) Differential expression of TMEM147 in the GDS4887 dataset.

(b) TMEM147 expression levels in HCC (n = 369) and non-tumor tissues (n = 160) in GEPIA database. The differential analysis is based on the selected datasets with TCGA tumors versus TCGA normal+GTEx normal. Data are presented as mean ± SEM. **P* < 0.01 (one-way ANOVA).

(c) Representative images of TMEM147 IHC staining in normal liver tissues, high grade HCC and low-grade HCC tissues, and percentage of patients with HCC histological differentiation. Scale bars, × 100: 200 μm; × 400: 50 μm.

(d) TMEM147 mRNA levels in HCC tissues and adjacent non-tumorous tissues.

(e) qRT-PCR analysis of TMEM147 expression in HCC cell lines.

(f) Western blot analysis of TMEM147 expression in HCC and matched non-tumor tissues.

(g) TMEM147 protein levels in HCC cell lines and relative band density.

(h) Kaplan–Meier overall survival and disease-free survival curves of patients with HCC with high (n = 182) and low (n = 182) expressions (stratified by median) of TMEM147 mRNAs in the GEPIA database using TCGA data (log-rank test).

(i) Kaplan–Meier survival analysis of patients with HCC according to TMEM147 expression.

All bar graphs are plotted as mean ± SEM of three independent experiments performed in triplicate. **P* < 0.05, ***P* < 0.01, ****P* < 0.001.

Abbreviations: TMEM147, Transmembrane protein 147; HCC, hepatocellular carcinoma; TCGA, The Cancer Genome Atlas; SEM, standard error of mean; ANOVA, analysis of variance; IHC, immunohistochemistry; qRT-PCR, quantitative real-time polymerase chain reaction

**Fig. 2 TMEM147 promotes HCC proliferation, invasion, and migration in vitro and in vivo.**

(a) Growth curve assay based on CCK8 analysis in HCC cells.

(b) Representative images of colony formation and statistical analysis of colony numbers.

(c and d) Representative images of Transwell migration and Matrigel invasion assay for the indicated cells. Scale bars, 100 μm.

(e) Representative images of subcutaneous xenograft derived from indicated HCC cells.

(f) Representative images of lung metastasis specimens of lung metastasis derived from tail injection with indicated cells, TMEM147 overexpression formed more and larger metastatic nodules, whereas TMEM147 knockdown displayed fewer and smaller nodules.

All experiments were performed three times and data are presented as mean ± SD. ***p*＜0.01; ****p*＜0.001.

Abbreviations: TMEM147, Transmembrane protein 147; HCC, hepatocellular carcinoma; CCK8, Cell counting Kit-8; SD, standard deviation; IHC, immunohistochemistry

**Fig. 3 TMEM147 upregulates 27HC by activating DHCR7, and promotes the progression of HCC**

(a) RNA-seq revealed the differentially regulated genes between TMEM147-silenced cells and control cells.

(b) Gene ontology (GO) analysis revealed that the differentially expressed genes were significantly enriched in cholesterol metabolism pathways.

(c) Protein levels of key enzymes in cholesterol metabolism pathways in TMEM147-silenced and control Huh7 cells.

(d) DHCR7 protein level of TMEM147 overexpression, silenced, and control cells.

(e) DHCR7 mRNA level of TMEM147 overexpression, silenced, and control cells.

(f) Cholesteryl esters (CEs) level of TMEM147 overexpression, silenced, and control HCC cells, and different TMEM147 expression subcutaneous tumors. Analyzed by ELISA assay.

(g) 27HC level of TMEM147 overexpression, silenced, and control HCC cells, and corresponding cell culture medium. Analyzed by ELISA assay.

(h) CYP27A1 protein level of TMEM147 overexpression, silenced, and control cells.

(i) Growth curve assay based on CCK8 analysis in TMEM147 overexpression HCC cells with different condition, including DHCR7 silencing, CYP27A1 inhibiting, and addition of exogenous 27HC.

(j) Representative images of Transwell migration and Matrigel invasion assay for the indicated cells. Scale bars, 100 μm.

Abbreviations: TMEM147, Transmembrane protein 147; HCC, hepatocellular carcinoma; 27HC, 27-hydroxycholesterol; DHCR7, 7-dehydrocholesterol reductase; IHC, immunohistochemistry; ELISA, enzyme-linked immunosorbent assay; CCK-8, cell counting kit-8.

**Fig. 4 TMEM147 promoting the expression of DHCR7 depends on transcription factor STAT2**

(a) TheTMEM147-interacting proteins were annotated with their Log_10_ ratio in Huh7 and HepG2 cell lysate.

(b) IP assay showed that Flag-TMEM147-antibody could pull down STAT2 (upper panel). Correspondingly, STAT2-antibody also pulled down TMEM1477 (lower panel).

(c) Schematic view of the putative binding sites and sequences of STAT2 on DHCR7 promoter region, predicted by the JASPAR and UCSC websites.

(d) STAT2 and p-STAT2 (Tyr690) protein levels in TMEM147 overexpression and silenced and control HCC cells.

(e) ChIP-qPCR assay was assessed using anti-STAT2 and anti-IgG antibody to identify STAT2 binding sites on the DHCR7 promoter in the indicated cells

(f) Luciferase reporter gene assays showed that binding of STAT2 to the wild-type DHCR7 promoter significantly increased the transcription of DHCR7.

(g) DHCR7 protein levels in STAT2-overexpressing, silenced, and control HCC cells.

(h) mRNA levels of TMEM147, DHCR7 and STAT2 relative to those of ACTIN were measured using RT-qPCR. Correlations among TMEM147, DHCR7 and STAT2 in HCC tumor are presented as Pearson’s correlation coefficients.

(i) Growth curve assay based on CCK8 analysis; interaction effects between si-STAT2 and TMEM147 on HCC cell proliferation.

(j) Representative images of Transwell assay; interaction effects between si-STAT2 and TMEM147 on HCC cell invasion and migration. Scale bars, 100 μm.

Abbreviations: TMEM147, Transmembrane protein 147; HCC, hepatocellular carcinoma; 27HC, 27-hydroxycholesterol; DHCR7, 7-dehydrocholesterol reductase; STAT2, signal transducer and activator of transcription 2; p-STAT2 (Tyr690), STAT2p phosphorylated at Tyr 690, IHC, immunohistochemistry; IP, immunoprecipitation; ChIP-qPCR, chromatin immunoprecipitation- quantitative real-time polymerase chain reaction.

**Fig. 5 TMEM147 upregulated 27HC and led to ferroptosis resistance by promoting GPX4 expression**

(a) GSH levels were assayed in the indicated erastin or RSL3 treated cells.

(b) MMP changes in the indicated erastin or RSL3 treated cells.

(c) Measurement of ROS in the indicated erastin or RSL3 treated cells. Scale bars, 50 μm.

(d) The indicated cells were treated with erastin or RSL3 and analyzed by TEM. Scale bars: 500 nm (rows 1 and 3) and 100 nm (rows 2 and 4).

(e) Representative images of subcutaneous xenograft derived from indicated HCC cells.

(f) Tumors were removed from mice and subjected to immunohistochemical staining with anti-4HNE antibodies. Representative images are shown. Scale bars: 50 μm.

(g) The iron metabolism-related protein level in the indicated cells.

(h) GPX4 protein levels in the indicated cells.

(i) GPX4 mRNA levels in the indicated cells.

Abbreviations: TMEM147, transmembrane protein 147; HCC, hepatocellular carcinoma; 27HC, 27-hydroxycholesterol; GPX4, glutathione peroxidase 4; GSH, glutathione; MMP, mitochondrial membrane potential; ROS, reactive oxygen species; TEM: transmission electron microscopy.

**Fig. 6 27HC enhances lipid metabolism in macrophages and activates M2 polarization**

(a) Schematic of conditional medium.

(b) Protein levels of M2 macrophage marker arginase 1 (ARG1) and CD206, FAO rate-limiting enzyme CPT1A and PPARγ in the indicated macrophages.

(c) Cell immunofluorescence of ARG1 in the indicated macrophages. Scale bars: 50 μm.

(d) Flow cytometry of CD163+ and HLA-DRα+ macrophages in different groups.

(e) Tumors were removed from mice and subjected to immunohistochemical staining with anti-ARG1 antibodies. Representative images are shown. Scale bars: 50 μm.

(f) Lipid content of the indicated macrophages. Analyzed by ELISA assay.

(g and h) Oxygen consumption rates (OCRs) of the indicated macrophages, and the statistic results depicting the basal and maximal respiration and spare respiratory capacity in the indicated macrophages by analyzing the OCRs. Statistical significance was determined by two-tailed Student’s *t* test between control MΦs and TAMs. *, *P* < 0.05; **, *P* < 0.01; ***, *P* < 0.001.

**Abbreviations:** 27HC, 27-hydroxycholesterol; FAO, fatty acid oxidation; PPAR, peroxisome proliferator-activated receptor.

**Fig. 7 27HC-Induced polarized macrophages enhance migration of HCC cells**

(a) The schematic of the co-culture method.

(b) Representative images of Transwell migration and Matrigel invasion assay for the indicated cells. Scale bars, 100 μm.

(c) Wound healing assay for the indicated cells. Scale bars, 100 μm.

(d) Representative images of lung metastasis specimens of lung metastasis derived from tail injection with indicated cells.

**Abbreviations:** 27HC, 27-hydroxycholesterol; HCC, hepatocellular carcinoma.

**Fig. S2**

1. qRT-PCR analysis of TMEM147 expression after lentiviral transfection.
2. Western blot analysis of TMEM147 expression after lentiviral transfection.

(c) Wound healing assays show that motility is enhanced in Huh7-TMEM147 cells, whereas TMEM147 knockdown suppresses motility in Huh7 and HepG2 cells.

Abbreviations: TMEM147, Transmembrane protein 147; qRT-PCR, quantitative real-time polymerase chain reaction

**Fig. S3**

(a) The mRNA level of key enzymes in cholesterol metabolism pathways in TMEM147-silenced and control Huh7 cells.

(b) CYP27A1 mRNA level of TMEM147 overexpression and silenced and control cells.

(c) qRT-PCR analysis of DHCR7 expression after siRNA transfection.

(d) DHCR7 protein level of the indicated conditions.

(e) Representative images of colony formation and statistical analysis of colony numbers.

(f) Wound healing assay for the indicated cells. Scale bars, 100 μm.

(g) Representative images of subcutaneous xenograft derived from indicated HCC cells.

(h) Representative images of lung metastasis specimens of lung metastasis derived from tail injection with indicated cells.

Abbreviations: TMEM147, Transmembrane protein 147; qRT-PCR, quantitative real-time polymerase chain reaction; DHCR7, 7-dehydrocholesterol reductase; HCC, hepatocellular carcinoma.

**Fig. S5**

(a) Ferrous ion (Fe^2+^) levels were assayed in in the indicated erastin or RSL3 treated cells.

(b) Mitochondrial malondialdehyde (MDA) levels were assayed in in the indicated erastin or RSL3 treated cells.

(c) mRNA levels of iron metabolism-related genes in the indicated cells.

**Fig. S6**

(a) mRNA levels of M2 macrophage marker arginase 1 (ARG1) and CD206, FAO rate-limiting enzyme CPT1A, and PPARγ in the indicated macrophages.

Abbreviations: FAO: fatty acid oxidation; PPAR, peroxisome proliferator-activated receptor.
