# Supplementary material for: TMEM147 aggravates the progression of HCC by modulating cholesterol homeostasis, suppressing ferroptosis, and promoting the M2 polarization of tumor-associated macrophages
Source: J Exp Clin Cancer Res. 2023 Oct 28;42:286. doi: 10.1186/s13046-023-02865-0 (PMC10612308; doi:10.1186/s13046-023-02865-0)
Supplement: Supplementary file 8 — Supplementary Material 8 [file 13046_2023_2865_MOESM8_ESM.docx]

October 4, 2023

Dear Editor,

Thanks very much for your kind work of our manuscript entitle “TMEM147 aggravates the progression of HCC by modulating cholesterol homeostasis, suppressing ferroptosis, and promoting M2 polarization of TAMs” (Manuscript ID: JECC-D-23-01357). On behalf of my co-authors, we would like to express our great appreciation to editor and reviewers.

We thank you and the reviewers for your thoughtful suggestions and insights. The manuscript has benefited from these insightful suggestions. I look forward to working with you and the reviewers to move this manuscript closer to publication in *Journal of Experimental & Clinical Cancer Research*. We provide a version with modified traces (manuscript-1) and a completed version (manuscript-2). Revised portion are marked in red in the paper. Meanwhile, the manuscript had be reviewed and edited by language services of ELSEVIER, and we provide a new certificate.

The responds to the comments are as following, the reviewer comments are laid out below in italicized font and specific concerns have been numbered. Our response is given in the blue text.

**Reviewer #1:**

*1. In the Background, the authors indicated that TMEM147 has been studied in various human diseases, including prostate cancer,[6] rheumatoid arthritis,[7] and colon cancer [8]. However, no any effect of TMEM147 on the other cancers is introduced, antitumorigenic or pro-tumorigenic?*

**Response:**

We feel great thanks for your professional review work on our article. According to your nice suggestions, we have made corrections to our previous draft, detailed corrections can be found on page 4, line190-193.

*2. Similarly, DHCR7 has vital functions in several malignant tumors, such as cervical [10], bladder,[11] ovarian,[12] gastric,[13] and colorectal 21 cancers [14]. It is very necessary to confirm its role of DHCR7 in the other cancers.*

**Response:**

Similarly, we think this is an excellent suggestion. We have re-written this part according to the reviewer's suggestion, page 5, line264-271 was added.

*3. The authors introduced that DHCR7 could be regulated by many* *transcription factors, such as sterol-regulatory element binding protein 1/2 (SREBP), [15, 16] osteogenic transcription factor (Runx2) [17] and E2F transcription factor 1 (E2F1) [18]. Did the authors probe the correlation of the above transcription factors with TMEM147?*

**Response:**

Thank you for pointing this out. As shown in Figure 3a, using co-IP assays and mass spectrometry analysis, we identified STAT2 instead of other transcription factors binding to TMEM147 in both Huh7 and HepG2 cell lines. In addition, DHCR7 being regulated by STAT2 has never been studied before, therefore it has a certain degree of innovation.

*4. The logicality of the whole manuscript is not very good. Why did the authors study TMEM147, 27HC, ferroptosis and M2 macrophage? and so on.*

**Response:**

Thank you very much for your question.

First, we searched the GEO database and TCGA transcriptome datasets and found that TMEM147 mRNA was significantly upregulated in HCC tissues. Meanwhile, Kaplan–Meier survival curve analysis of TCGA datasets demonstrated that HCC patients with increased TMEM147 protein expression had poorer overall five-year survival. Therefore, we chose TMEM147 as the target gene for our study.

Next, previous literature has confirmed that TMEM147 can regulate DHCR7 expression and TMEM147-DHCR7 pathway can promote cholesterol accumulation.^1^ A study showed that, in breast cancer, cholesterol mediates the metastatic effects via its oxysterol metabolite, 27-hydroxycholesterol.^2^ Therefore, we suppose that cholesterol accumulation caused by TMEM147-DHCR7 may also cause an increase in 27HC in HCC. 27HC plays a promoting role in various cancers,^2-4^ so we speculate that 27HC also promotes HCC progression.

Later, previous studies have shown that chronic exposure to 27HC increases lipid accumulation in cells and enhances the resistance of breast cancer cells to ferroptosis, whereas GPX4 inhibition reverses this effect.^5^ There are also studies have shown that pretreatment of macrophages with 27HC can inhibit T cell function.^3^ However, it remains unclear whether tumor-associated macrophages, which are the most abundant immune cells in the tumor microenvironment, are also regulated by 27HC. As mentioned above, we explored the effects of 27HC on ferroptosis and tumor-associated macrophages.

*5.What kinds of mice were used in this study?*

**Response:**

Thank you very much for your suggestion. We have added the detailed information of mice. Detailed corrections can be found on page 15, line798-800.

*6. What are the mechanisms of TMEM147 boosting tumor cell proliferation and metastasis? ferroptosis or M2 macrophage?*

**Response:**

Thanks very much for your comment. Our results confirm that TMEM147 mediates the progression of HCC through TMEM147/STAT2/DHCR7/27HC pathway. 27HC can be discharged into the TME and promoting M2 polarization, then promotes HCC invasion and metastasis. Furthermore, we have also identified that 27HC induces ferroptosis resistance. In brief, 27HC has been identified as a key downstream target of TMEM147 to induce tumor cell growth and metastasis, ferroptosis resistance, and M2 polarization of TAMs. Of course, it cannot be excluded that 27HC might promote the progression of HCC through other pathways, moreover, the downstream cancer promoting mechanism of 27HC needs further exploration. We have carefully considered this suggestion of reviewer and make some changes, detailed corrections can be found on page 25, line1203-1206; page28, line1344-1347; and the figure 8. The modified Figure 8 is shown below

**Reviewer #2:**

*1. In the introduction, line 5, it was written* *" its role in HCC has not been explored in HCC" Please remove one of the" in HCC".*

**Response:**

We sincerely thank the reviewer for careful reading. As suggested by the reviewer, we have corrected the “its role in HCC has not been explored in HCC” into “its role in HCC has not been explored”

*2. Which type of primary and secondary antibodies were used in immunohistochemistry? some details in the experiment should be written*

**Response:**

Thank you very much for your suggestion. We have added the detailed information of primary and secondary antibodies used in immunohistochemistry. Detailed corrections can be found on page 8, line561-562.

*3. In "chromatin immunoprecipitation", it was written "formaldehyde solution" Please rewrite using "**formalin solution"*

**Response:**

Thanks for your careful checks. We are sorry for our carelessness. Based on your comments, we have made the correction: “formaldehyde solution” into “formalin solution”.

*4. Authors didn't determine the role of TMEM147* *in angiogenesis*

**Response:**

We appreciate the reviewer’s insightful suggestion and agree that it would be useful to demonstrate more comprehensive functions of TMEM147 in HCC. In this research, we are more focused on cholesterol homeostasis and tumor immune microenvironment. We did not conduct the experiment of angiogenesis due to funding and time constraints, and we will consider this direction in our future research.

*5. The limitation was not written. Authors should observe the limitations of the study*

**Response:**

Thank you very much for pointing this out. Our research only studied the effect of TMEM147 on tumor associated macrophages in the immune microenvironment, in the future, we should further explore the regulatory effects of TMEM147 on other immune cells. Additionally, whether TMEM147 can promote HCC through other molecular pathways besides DHCR7 needs further research.

*6. English Typos should have been revised thoroughly over all the text.*

**Response:**

Thanks for your careful checks. Based on your comments, the typo is revised in our resubmitted manuscript, and the manuscript has been carefully revised by language editing service again to improve the grammar and readability.

Thank you for your consideration. I look forward to hearing from you.

Sincerely,

Professor Wenjie Dai

The First Affiliated Hospital of Harbin Medical University

Harbin, Heilongjiang Province, 150001, People's Republic of China.

daiwenjiehmu@163.com

Reference

1. A. Christodoulou, G. Maimaris, A. Makrigiorgi, E. Charidemou, C. Lüchtenborg, A. Ververis, R. Georgiou, C. Lederer, C. Haffner, B. Brügger , N. Santama. TMEM147 interacts with lamin B receptor, regulates its localization and levels, and affects cholesterol homeostasis. Journal of cell science. 133, (2020).

2. A. Baek, Y. Yu, S. He, S. Wardell, C. Chang, S. Kwon, R. Pillai, H. McDowell, J. Thompson, L. Dubois, P. Sullivan, J. Kemper, M. Gunn, D. McDonnell , E. Nelson. The cholesterol metabolite 27 hydroxycholesterol facilitates breast cancer metastasis through its actions on immune cells. Nature communications. 8, 864 (2017).

3. L. Ma, L. Wang, A. Nelson, C. Han, S. He, M. Henn, K. Menon, J. Chen, A. Baek, A. Vardanyan, S. Shahoei, S. Park, D. Shapiro, S. Nanjappa , E. Nelson. 27-Hydroxycholesterol acts on myeloid immune cells to induce T cell dysfunction, promoting breast cancer progression. Cancer letters. 493, 266-283 (2020).

4. S. Hiramitsu, T. Ishikawa, W. Lee, T. Khan, C. Crumbley, N. Khwaja, F. Zamanian, A. Asghari, M. Sen, Y. Zhang, J. Hawse, J. Minna , M. Umetani. Estrogen Receptor Beta-Mediated Modulation of Lung Cancer Cell Proliferation by 27-Hydroxycholesterol. Frontiers in endocrinology. 9, 470 (2018).

5. W. Liu, B. Chakraborty, R. Safi, D. Kazmin, C. Y. Chang , D. P. McDonnell. Dysregulated cholesterol homeostasis results in resistance to ferroptosis increasing tumorigenicity and metastasis in cancer. Nat Commun. 12, 5103 (2021).
